# Both Hypoxia-Inducible Factor 1 and MAPK Signaling Pathway Attenuate PI3K/AKT via Suppression of Reactive Oxygen Species in Human Pluripotent Stem Cells

**DOI:** 10.3389/fcell.2020.607444

**Published:** 2021-01-21

**Authors:** Petr Fojtík, Deborah Beckerová, Katerina Holomková, Martin Šenfluk, Vladimir Rotrekl

**Affiliations:** ^1^Department of Biology, Faculty of Medicine, Masaryk University, Brno, Czechia; ^2^International Clinical Research Center (ICRC), St. Anne's University Hospital, Brno, Czechia

**Keywords:** PI3K/AKT, MAPK, reactive oxygen species, hPSCs, HIF-1, hypoxia

## Abstract

Mild hypoxia (5% O_2_) as well as FGFR1-induced activation of phosphatidylinositol-4,5-bisphosphate 3-kinase/protein kinase B (PI3K/AKT) and MAPK signaling pathways markedly support pluripotency in human pluripotent stem cells (hPSCs). This study demonstrates that the pluripotency-promoting PI3K/AKT signaling pathway is surprisingly attenuated in mild hypoxia compared to the 21% O_2_ environment. Hypoxia is known to be associated with lower levels of reactive oxygen species (ROS), which are recognized as intracellular second messengers capable of upregulating the PI3K/AKT signaling pathway. Our data denote that ROS downregulation results in pluripotency upregulation and PI3K/AKT attenuation in a hypoxia-inducible factor 1 (HIF-1)-dependent manner in hPSCs. Using specific MAPK inhibitors, we show that the MAPK pathway also downregulates ROS and therefore attenuates the PI3K/AKT signaling—this represents a novel interaction between these signaling pathways. This inhibition of ROS initiated by MEK1/2–ERK1/2 may serve as a negative feedback loop from the MAPK pathway toward FGFR1 and PI3K/AKT activation. We further describe the molecular mechanism resulting in PI3K/AKT upregulation in hPSCs—ROS inhibit the PI3K's primary antagonist PTEN and upregulate FGFR1 phosphorylation. These novel regulatory circuits utilizing ROS as second messengers may contribute to the development of enhanced cultivation and differentiation protocols for hPSCs. Since the PI3K/AKT pathway often undergoes an oncogenic transformation, our data could also provide new insights into the regulation of cancer stem cell signaling.

## Introduction

Human pluripotent stem cells (hPSCs) hold great promise for disease modeling, development of cell replacement therapies, and human embryology research due to their unique properties such as self-renewal and pluripotency. Traditionally, pluripotent stem cells can be divided into two distinct categories with very similar gene expression profiles: embryonic stem cells (ESCs) and induced pluripotent stem cells (iPSCs). While ESCs can be derived directly from preimplantation blastocysts, iPSCs are created by reprogramming somatic cells (Takahashi et al., 2007). Preimplantation blastocysts used for the preparation of ESCs naturally reside in a low oxygen environment (Fischer and Bavister, 1993; Okazaki and Maltepe, 2006). It has been shown that low oxygen conditions prevent differentiation and support pluripotency in various stem cell populations (Ezashi et al., 2005; Forristal et al., 2010) and enhance the generation of human iPSCs from fibroblasts (Yoshida et al., 2009). Mathieu et al. (2013) have reported that the transition of committed cells to hypoxic conditions can reverse the differentiation commitment back to full pluripotency. Despite these findings, hPSCs are commonly maintained in a 21% O_2_ (atmospheric oxygen concentration), although hypoxic conditions would seem more physiological (for the purposes of this publication, we will further refer to 5% O_2_ as mild hypoxia). In order to maintain hPSCs self-renewal and pluripotency *in vitro*, fibroblast growth factor 2 (FGF2) is commonly used (Xu et al., 2005b; Levenstein et al., 2006; Eiselleova et al., 2009). Upon binding to fibroblast growth factor receptor (FGFR), FGF2 activates MAPK, phosphatidylinositol-4,5-bisphosphate 3-kinase/protein kinase B (PI3K/AKT), phospholipase Cγ as well as the Janus kinase/signal transducers and activators of transcription pathways.

Two of these pathways, MAPK and PI3K/AKT, have been implicated to play a role in pluripotency maintenance, self-renewal, reprogramming, and cell fate decision (Armstrong et al., 2006; Li et al., 2007; Yu et al., 2011; Wang et al., 2017; Haghighi et al., 2018), and their context-dependent crosstalk (Mendoza et al., 2011; Aksamitiene et al., 2012) contribute to the regulation of these processes (Dalton, 2013). The hypoxic environment was shown to enhance MAPK (Miyamoto et al., 2015) and PI3K/AKT pathway activity in many cell types and therefore contributes to angiogenesis (Shiojima and Walsh, 2002; Hung et al., 2007; Zhang et al., 2011), promotes survival/prevents apoptosis (Alvarez-Tejado et al., 2001; Lee et al., 2013), and stimulates glycolytic enzyme upregulation as part of the cell's adaptation process to the hypoxic conditions (Ward and Thompson, 2012; Zeng et al., 2016). A recent study performed on mouse ESCs (mESCs) showed that hypoxic conditions lead to downregulation of both the MAPK and the PI3K/AKT signaling and suggested that this effect is secured by hypoxia-mediated downregulation of reactive oxygen species (ROS) (Kučera et al., 2017). At physiological levels, ROS act as signaling molecules capable of regulating various intracellular pathways. ROS-mediated changes in cellular signaling have also been described to affect cell fate decision (Rhee, 2006; Genestra, 2007; Zhang and Yang, 2013; Khacho et al., 2016; Zhang et al., 2016; Kim et al., 2018). It should be noted that ROS levels have been described to be pathologically elevated in some of the iPSCs disease models, where they could affect differentiation (Jelinkova et al., 2019).

To fully utilize the hPSCs potential of self-renewal and differentiation into the desired cell types, it is essential to understand the signaling pathways driving cell fate decision. Since hypoxic conditions support pluripotency maintenance (Mathieu et al., 2013), we investigated whether mild hypoxia modulates FGF2 signaling pathways and their crosstalk in hPSCs. Our data suggest that ROS serve as second messengers activating PI3K/AKT. To the best of our knowledge, we are the first to report that ROS downregulation caused by mild hypoxia and MAPK activation attenuates the pluripotency-maintaining PI3K/AKT signaling in hPSCs.

## Materials and Methods

### Culture

Experiments were performed using human hESCs line CCTL12 and CCTL14 and human iPSCs line AM13, previously characterized by Adewumi et al. (2007) and Krutá et al. (2014). Long-term cultivation of hPSCs was performed on mitotically inactivated mouse embryonic fibroblast (MEF) from the CD1 and CF1 mouse strain in human embryonic stem cell media (hES) containing Dulbecco's modified Eagle medium (DMEM)/F12 (Thermo Fisher Scientific, Waltham, MA, USA, 21331-020) supplemented with 15% (vol/vol) knockout serum replacement (Thermo Fisher Scientific, 10828-028), non-essential amino acids (Thermo Fisher Scientific, 11140-035), 4 ng/ml FGF-2 (PeproTech, Cranbury, NJ, USA, 100-18B), l-glutamine, 0.5% (vol/vol; Biosera, Boussens, France, XC-T1715), penicillin–streptomycin (Biosera, XC-A4122), and 2-mercaptoethanol (Sigma-Aldrich, St. Louis, MO, USA, M3148) in a colony type culture. For experimental procedures, hPSCs were cultured feeder free on Matrigel hESC-qualified Matrix (Matrigel; Corning, NY, USA, 354277) coated dishes and cultivated in MEF-conditioned hES media (CM+) supplemented with FGF2 (10 ng/ml) in a monolayer-type culture. hES media was conditioned on mitotically inactivated MEF for 24 h, and the same dish containing MEF was used seven times; then, all seven batches were mixed, supplemented with l-glutamine (0.5% vol/vol) and FGF2 (10 ng/ml) and filtered. MEF-conditioned media without FGF2 (CM–) was prepared using hES media missing FGF2, and the CM– was not further supplemented with FGF2. hPSCs maintained on Matrigel-coated dishes were cultivated for a maximum of seven passages. For cultivation in 5% O_2_, cells were kept in the MCO-18M multigas incubator (Sanyo, Moriguchi, Osaka, Japan). PD184352 (PD18; 25 μM; Sigma-Aldrich, PZ0181) and PD0325901 (PD03; 0.2 μM; Sigma-Aldrich, PZ0162) were used to inhibit MEK1/2. Wortmannin (WRT; 1 μM; Sigma-Aldrich, W1628) was used to inhibit PI3K; CoCl_2_ (50 μM; Sigma-Aldrich, 60818) was used to stabilize α subunits of hypoxia-inducible factors. AS1938909 (AS19; 10 mM; Calbiochem, San Diego, CA, USA, 565840) was used to inhibit SHIP2. Okadaic acid (OKA; 0.5 and 10 nM; Sigma-Aldrich, O9381) was used to inhibit PP2A. GSH (10 mM; Sigma-Aldrich, G6013) was used to downregulate ROS, and H_2_O_2_ (0.5 mM; Sigma-Aldrich, H1009) was used to induce ROS. Endoribonuclease-prepared short-interfering RNAs (esiRNAs) against hypoxia-inducible factor 1α (HIF-1α; 50 pmol; Sigma-Aldrich, EHU151981) and PTEN (50 pmol; Sigma-Aldrich, EHU106441) were introduced into the cells using Lipofectamine 2000 Transfection Reagent (Thermo Fisher Scientific, 11668-037) according to the manufacturer's manual to downregulate HIF-1α (an oxygen-sensitive subunit of HIF1) and PTEN (antagonist of PI3K). Successful downregulation (48 h after transfection) was confirmed using Western blot (WB).

### Western Blotting

hPSCs cultivated on Matrigel-coated dishes to the maximum of 70% confluence were washed three times with 1× phosphate-buffered saline (PBS) and lysed on ice with 1% sodium dodecyl sulfate (SDS) lysis buffer (50 mM Tris–HCl, 1% SDS, pH 6.8). Protein concentrations were quantified using the DC Protein Assay (Bio-Rad, Hercules, CA, USA, 5000111) measured in triplicates on DTX 880 Multimode Detector (Beckman Coulter, Brea, CA, USA). Protein concentrations were then adjusted to 1 mg/ml, 10× Laemmli buffer was added, and the lysates were briefly boiled. In cases where protein concentration adjustment was unnecessary, hPSCs were lysed directly with 2× Laemmli buffer after the 1× PBS wash. SDS–polyacrylamide gel electrophoresis (SDS-PAGE) was performed using either 8 or 10% polyacrylamide gels, 10–20 μg of total protein was loaded, and gel electrophoresis was ran at 140 V/70 min. Proteins were then transferred to Immobilon-P polyvinylidene fluoride (PVDF) membrane (Merck Millipore, Burlington, MA, USA, IPVH00010) 100 V/60 min.

The membranes were blocked in 5% dried milk or bovine serum albumin in Tris-buffered saline containing 0.1% Tween-20 (TBS-T) for 1 h and incubated overnight with antibodies diluted in the respective blocking solutions at 4°C. The following day, membranes were washed 3 × 15 min with TBS-T and incubated with secondary antibodies diluted in respective blocking solution for 1 h at room temperature followed by 5 × 10 min washes with TBS-T. Immobilon Western Chemiluminescent HRP Substrate (Merck Millipore, P90720) was used as a substrate for the luminescence reaction. Developing was done in G:Box Chemi (SYNGENE, Bangalore, India). Obtained images were adjusted using the GIMP2 software and analyzed in ImageJ. Uncropped blots with molecular markers are shown in Raw Images 1.

For the detection of PTEN, redox state cells were harvested in native lysis buffer [100 mM Tris pH 7, 150 mM NaCl, 1 mM ethylenediaminetetraacetic acid (EDTA), 0.1% Triton X-100] supplemented with the cOmplete Mini Protease Inhibitor Cocktail (Roche, Basel, Switzerland; 11836153001) and 50 mM N-ethylmaleimide (Sigma-Aldrich; 04259). Samples were sonicated, mixed with a non-reducing loading buffer, and resolved using 8% SDS-PAGE at 4°C. Protein transfer and detection were performed as described above.

WB quantification analysis was performed using the ImageJ software. Mean gray values for individual proteins were measured using rectangle selection with a diameter fitted tightly to the largest band of that specific protein in a given WB. The same-sized rectangle selection was used for the measurement of all specific protein bands in the lane. Eventual background was subtracted using the same selection rectangle next to the specific protein band. Proteins of interest were then normalized to a loading control. Values chosen for relativization are indicated in the individual figure legends.

The following primary antibodies were used for WB: anti-ERK1/2 (9102), anti-pERK1/2 (9101S), anti-AKT (9272), anti-pAKT (9271), anti-HIF-1α (3716), anti-β-actin (3700), anti-FGFR1 (9740), anti-pFGFR1 (3476), anti-PTEN (9188), anti-pPTEN (9554), anti-PI3K p85 (4292), anti-pPI3K p85/55 (4228) (Cell Signaling Technology, Danvers, MA, USA), anti-α-Tubulin (Exbio, Prague, Czech Republic, 11-250-C100), anti-Lamin B1 (Santa Cruz Biotechnology, Dallas, TX, USA, sc-374015), and anti-PCNA (Sigma-Aldrich, HPA030522). We used the following secondary antibodies: antirabbit immunoglobulin G–horse radish peroxidase (IgG-HRP) (Cell Signaling Technology, 7074) and goat antimouse IgG-HRP (Merck Millipore, 12-349).

### Analysis of Reactive Oxygen Species Generation

Cells grown on Matrigel-coated coverslips were treated with the above-described substances. Fifty minutes before the end of the treatment, CellROX Green (5 mM; Thermo Fisher Scientific, C10444) was added. Afterward, the cells were washed three times in PBS on ice and fixed with 4% paraformaldehyde (Sigma-Aldrich, 158127) for 30 min. Snapshots of the approximately same-sized cell clusters were taken with constant exposure time using a fluorescent LSM700 microscope (×40 1.3 oil differential interference contrast objective; Carl Zeiss, Oberkochen, Germany) within 6 h of fixation. ROS levels were determined as fluorescence divided by the fluorescence area in raw images using the ImageJ software.

### ICC Analysis

Cells cultivated on Matrigel-coated coverslips were washed three times with PBS, fixed with 4% paraformaldehyde (Sigma-Aldrich, 158127) for 30 min, and blocked with 1% bovine serum albumin (BSA) in 0.1% Triton X-100 in PBS for 1 h. Coverslips were then incubated with a rabbit polyclonal anti-Nanog antibody (Santa Cruz Biotechnology, sc-33759) and a mouse polyclonal anti-Oct-3/4 (Santa Cruz Biotechnology, sc-5279) at a dilution of 1:200 overnight at 4°C. Donkey antirabbit Alexa 594 (Thermo Fisher Scientific, A21207) and donkey antimouse Alexa 488 (Thermo Fisher Scientific, A21202) were used as secondary antibodies at a dilution of 1:500 at room temperature for 1 h. Nuclei were counterstained using 4′,6-diamidino-2-phenylindole (DAPI). Snapshots were taken with constant exposure time for individual channels using a fluorescent LSM700 microscope (×63 oil immersion objective; Carl Zeiss). Signal density per area was calculated for individual nuclei from raw images using ImageJ, and mitotic, overlapping, or otherwise irregular nuclei were left out of the analysis.

### RNA Isolation and Quantitative Real-Time PCR

Total RNA was isolated using the RNA Blue reagent (Top-Bio, Czech Republic) according to the manufacturer's protocol. Messenger RNA (mRNA) concentration and purity were determined using NanoDrop (NanoDrop Technologies, Wilmington, Germany). Two micrograms of total RNA were transcribed into complementary DNA (cDNA) using the Moloney Mouse Leukemia Virus (M-MLV) reverse transcriptase (Invitrogen, Carlsbad, CA, USA) and Oligo(dT) primers (Thermo Fisher Scientific Inc., USA) at 37°C for 1 h followed by 5 min at 85°C. Quantitative real-time PCR (qRT-PCR) was performed using the LightCycler^®^480 DNA SYBR Green I Master (Roche) in a Light Cycler 480 instrument. The obtained data were normalized to glyceraldehyde 3-phosphate dehydrogenase (GAPDH) mRNA expression and are presented as 2–Δcq. Sequences of the primers used were as follows: GAPDH (forward: AGCCACATCGCTCAGACACC; reverse: GTACTCAGCGCCAGCATCG), POU5F1 (forward: GCAAAGCAGAAACCCTCGT; reverse: ACACTCGGACCACATCCTTC), Sox2 (forward: ATGCACCGCTACGACGTGA; reverse: CTTTTGCACCCCTCCCATTT), and Nanog (forward: CCTATGCCTGTGATTTGTGG, reverse: CTGGGACCTTGTCTTCCTTT).

### Statistical Analysis

The number of independent experiments is indicated in the figure legends. Arithmetical means and SEM/SD were calculated using the GraphPad Prism 8 software (GraphPad Software, La Jolla, CA, USA). Statistical significance was determined using a two-tailed Mann–Whitney test, one-sample Student's *t-*test, or a paired two-tailed Student's *t-*test. Asterisks denote a significant difference as follows: ^*^p < 0.05; ^**^p < 0.01; ^***^p < 0.0001. Source data used to generate the graphs are shown in Supplementary Data 1.

## Results

### Mild Hypoxia Downregulates PI3K/AKT via ROS Attenuation

**Figure 1:**
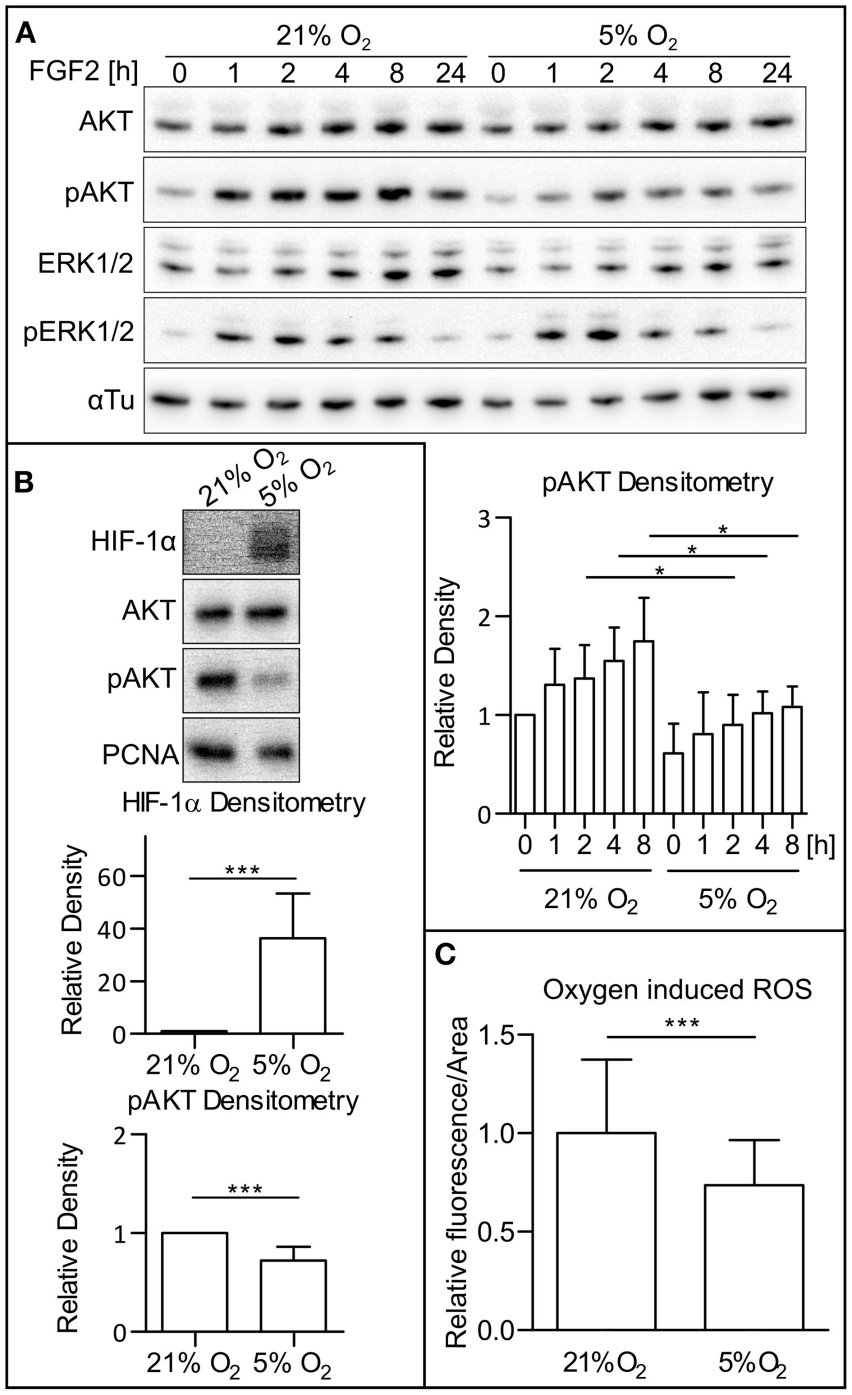
Low O_2_ (5%) decreases AKT phosphorylation and ROS levels. **(A)** WB analysis of AKT and ERK1/2 phosphorylation in 21 and 5% O_2_ in CCTL14 hESCs and pAKT quantification. Cells kept in 21 or 5% O_2_ were starved for 24 h and subsequently treated with FGF2 (10 ng/ml); samples were then collected at different time points. Alpha tubulin was used as a loading control. Data in the graph are presented as mean ± SD (21% O_2_: 0 h, 1 ± 0, *n* = 4; 1 h, 1.307 ± 0.364, *n* = 4; 2 h, 1.373 ± 0.338, *n* = 4; 4 h, 1.548 ± 0.340, *n* = 4; 8 h, 1.747 ± 0.444, *n* = 4; 5% O_2_: 0 h, 0.611 ± 0.301, *n* = 4; 1 h, 0.809 ± 0.421, *n* = 4; 2 h, 0.9 ± 0.306, *n* = 4; 4 h, 1.019 ± 0.220, *n* = 4; 8 h, 1.082 ± 0.208, *n* = 4). Statistical analysis was conducted using paired two-tailed *t-*test with statistical significance taken where *p < 0.05. **(B)** Analysis of HIF-1α and pAKT levels in 21 and 5% O_2_ in hPSCs. Cells cultivated for 24 h in 21 and 5% O_2_ were collected 1–2 h after the treatment with FGF2. PCNA was used as a loading control. Data in graphs are presented as mean ± SD (HIF-1α: 21% O_2_, 1 ± 0, *n* = 11; 5% O_2_, 36.26 ± 17.09, *n* = 11; pAKT: 21% O_2_, 1 ± 0, *n* = 26; 5% O_2_, 0.721 ± 0.141, *n* = 26). Statistical analysis was conducted using one sample *t-*test (theoretical mean = 1) with statistical significance taken where ***p < 0.0001. **(C)** Analysis of ROS level in CCTL14 kept in 21 and 5% O_2_ environment. Cells were cultivated in respective oxygen concentrations for 24 h, followed by a change of media and additional 2-h cultivation. Normalized data from five different experiments (with at least 10 measurements in each) are presented as mean ± SD (21% O_2_, 1 ± 0.0491, *n* = 58; 5% O_2_, 0.7411 ± 0.0299, *n* = 58). Statistical significance was calculated using a two-tailed Mann–Whitney test (***p < 0.0001). Please refer to Raw Images 1 for uncropped WB and to Supplementary Data 1 for source data used to generate the graph shown in the figure. WB, western blot; ROS, reactive oxygen species; CM+, conditioned media with FGF2 (10 ng/ml); FGF2, fibroblast growth factor 2; AKT, protein kinase B; pAKT, phosphorylated protein kinase B; ERK1/2, extracellular signal-regulated kinase 1 and 2; pERK1/2, phosphorylated extracellular signal-regulated kinase 1 and 2; αTu, alpha tubulin.

To determine the dependency of PI3K/AKT and MAPK signaling dynamics on oxygen level, hPSCs were cultivated under both atmospheric oxygen (21% O_2_) and mild hypoxic (5% O_2_) conditions. Western blot analysis of cell lysates revealed that the amount of active (phosphorylated) AKT is downregulated upon FGF2 administration (10 ng/ml) under mildly hypoxic conditions when compared to 21% O_2_ conditions and that this effect persists in time. It ought to be noted that no difference was observed under FGF2 deprivation. We also did not observe a significant difference in extracellular signal-regulated kinase 1 and 2 (ERK1/2) phosphorylation when comparing cells cultivated under 21% O_2_ and 5% O_2_ over the 24-h period (Figure 1A). This effect was observed in two hESC lines (CCTL14 and CCTL12; Figure 1A and Supplementary Figure 1A) and one iPSC line (AM13; Supplementary Figure 1B). Based on these results, further experiments were conducted 1 or 2 h after the FGF2 treatment. We noted a significant decrease in AKT phosphorylation and stabilization of HIF-1α in 5% O_2_ (Figure 1B).

It has been previously established that hypoxic conditions lead to lower ROS levels in mammalian cultures (Maddalena et al., 2017). To confirm this observation in our model, we cultivated hPSCs in 21 and 5% O_2_ and compared ROS levels using a ROS-sensitive fluorescent probe in combination with cell imaging. Compared to 21% O_2_, the levels of ROS in CCTL14 cells cultivated in mild hypoxia were significantly decreased (Figure 1C).

**Figure 2:**
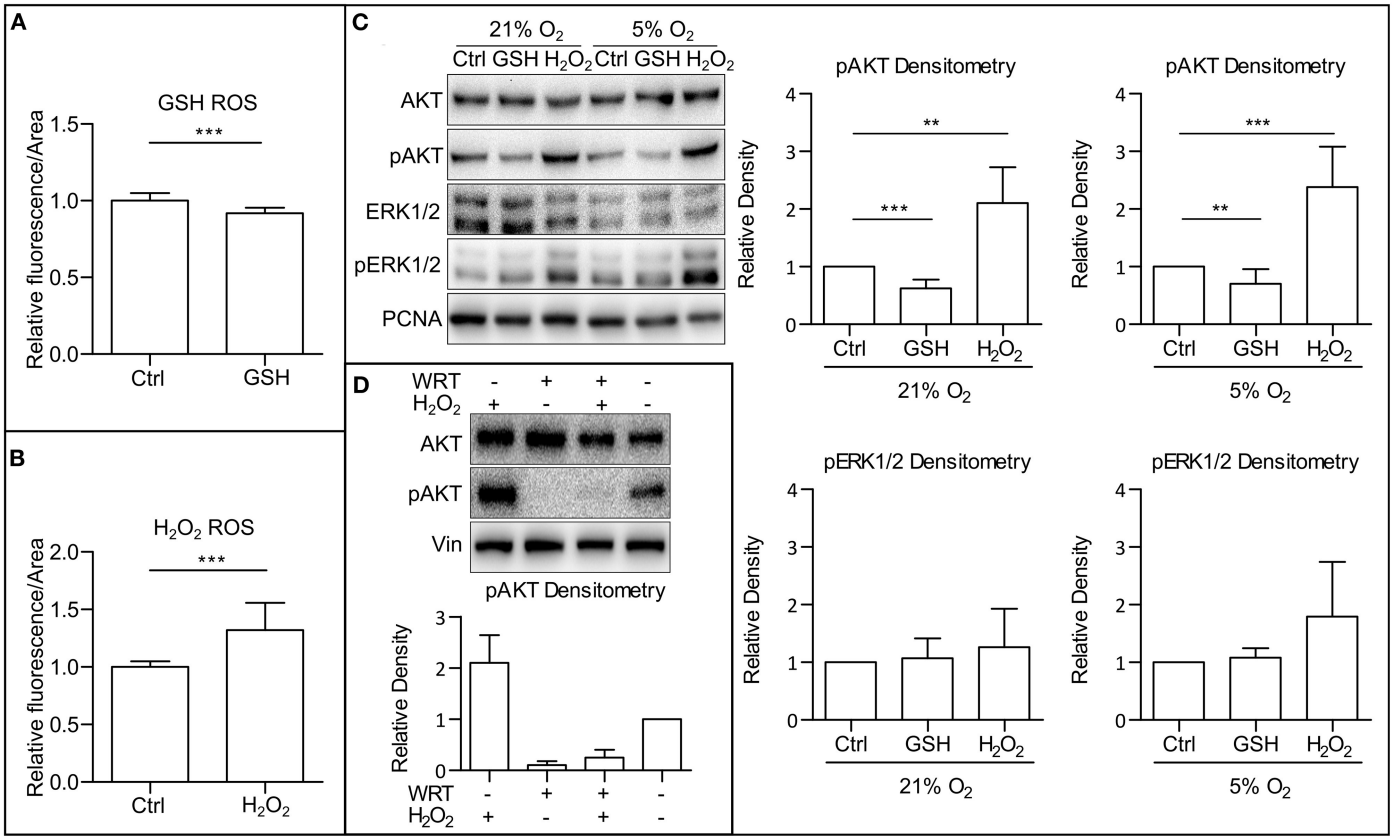
ROS upregulate AKT phosphorylation. **(A)** Downregulation of ROS by GSH in CCTL14 hESCs. Cells in CM+ were treated for 1 h with 10 mM GSH in 5% O_2_ after media change. Normalized data from three different experiments (with at least 10 individual measurements in each) are presented as mean ± SD (Ctrl, 1 ± 0.0490, *n* = 33; GSH, 0.9187 ± 0.0349, *n* = 31). Statistical significance was calculated using a two-tailed Mann–Whitney test (***p < 0.0001). **(B)** Upregulation of ROS by H_2_O_2_ in CCTL14 hESCs. Cells in CM+ were treated for 1 h with 0.5 mM H_2_O_2_ in 5% O_2_ after media change. Normalized data from three different experiments (with at least 10 individual measurements in each) are presented as mean ± SD (Ctrl, 1 ± 0.0088, *n* = 32; H_2_O_2_, 1.321 ± 0.0404, *n* = 34). Statistical significance was calculated using a two-tailed Mann–Whitney test (***p < 0.0001). **(C)** WB analysis of ROS-induced changes in AKT and ERK1/2 phosphorylation and their quantification in hPSCs. Cells were cultivated in CM+ for 24 h in 21 and 5% O_2_, respectively, then treated with 10 mM GSH and 0.5 mM H_2_O_2_ for 1 h in fresh media. PCNA was used as a loading control. Data in graphs are presented as mean ± SD (pAKT in 21% O_2_: Ctrl, 1 ± 0; GSH, 0.622 ± 0.155, *n* = 10; H_2_O_2_, 2.105 ± 0.621, *n* = 6; pAKT in 5% O_2_: Ctrl, 1 ± 0; GSH, 0.703 ± 0.257, *n* = 9; H_2_O_2_, 2.379 ± 0.703, *n* = 8; pERK1/2 in 21% O_2_: Ctrl, 1 ± 0; GSH, 1.069 ± 0.344, *n* = 8; H_2_O_2_, 1.260 ± 0.667, *n* = 6; pERK1/2 in 5% O_2_: Ctrl, 1 ± 0; GSH, 1.083 ± 0.160, *n* = 8; H_2_O_2_, 1.793 ± 0.952, *n* = 6). Statistical analysis was conducted using one sample *t-*test (theoretical mean = 1) with statistical significance taken where **p < 0.01, ***p < 0.001. **(D)** WB analysis of PI3K-independent effect of ROS on AKT phosphorylation. PI3K activity was inhibited using wortmannin (WRT, 1 μM/1 h) and ROS were induced using H_2_O_2_ (1 mM/1 h). Data in graph are presented as mean ± SEM (–/H_2_O_2_, 2.10 ± 0.54, *n* = 4; WRT/–, 0.107 ± 0.071, *n* = 4; WRT/H_2_O_2_, 0.149 ± 0.299, *n* = 4; –/–, 1 ± 0). Please refer to Raw Images 1 for uncropped WB and to Supplementary Data 1 for source data used to generate the graphs shown in the figure. WB, western blot; ROS, reactive oxygen species; GSH, glutathione; Ctrl, control; CM+, conditioned media with FGF2 (10 ng/ml); AKT, protein kinase B; pAKT, phosphorylated protein kinase B; ERK1/2, extracellular signal-regulated kinase 1 and 2; pERK1/2, phosphorylated extracellular signal-regulated kinase 1 and 2; PCNA, proliferating cell nuclear antigen; WRT, wortmannin.

ROS are known to directly affect signaling pathways (Zhang et al., 2016). Reports describe positive effects of ROS on the activity of PI3K/AKT (Okoh et al., 2013; Zhang and Yang, 2013). After establishing that mild hypoxia modulates both ROS levels (Figure 1C) and AKT phosphorylation (Figures 1A,B and Supplementary Figures 1A,B) in hPSCs, we wanted to study whether it is ROS that mediate AKT phosphorylation. To study this hypothesis, we used 10 mM GSH to downregulate and 0.5 mM H_2_O_2_ to upregulate ROS levels (Figures 2A,B) and analyzed their effect on AKT and ERK1/2 phosphorylation in different oxygen concentrations. Both treatments were applied 1 h prior to sample collection. GSH managed to downregulate the levels of phosphorylated AKT in hPSCs grown in 21% O_2_ to levels similar to mild hypoxia, while no effect on ERK1/2 phosphorylation was observed (Figure 2C). The upregulation of ROS by H_2_O_2_ led to an increase in both AKT and statistically insignificantly in ERK1/2 phosphorylation (Figure 2C) under mildly hypoxic as well as atmospheric oxygen concentrations. The effect of ROS modulation on AKT phosphorylation was also observed in the CCTL12 hESC line (Supplementary Figure 2A) and AM13 iPSC line (Supplementary Figure 2B). The correlation between ROS levels (driven by oxygen tension or induced by H_2_O_2_) and AKT phosphorylation suggests that mild hypoxia downregulates AKT phosphorylation and thus PI3K/AKT pathway activity *via* modulation of ROS as second messengers in hPSCs. To determine whether ROS can induce AKT phosphorylation independently of PI3K, the cells were treated with a combination of compounds—H_2_O_2_ and a PI3K inhibitor wortmannin (WRT). The results confirm that ROS-mediated increase in AKT phosphorylation is dependent on PI3K activity (Figure 2D).

**Figure 3:**
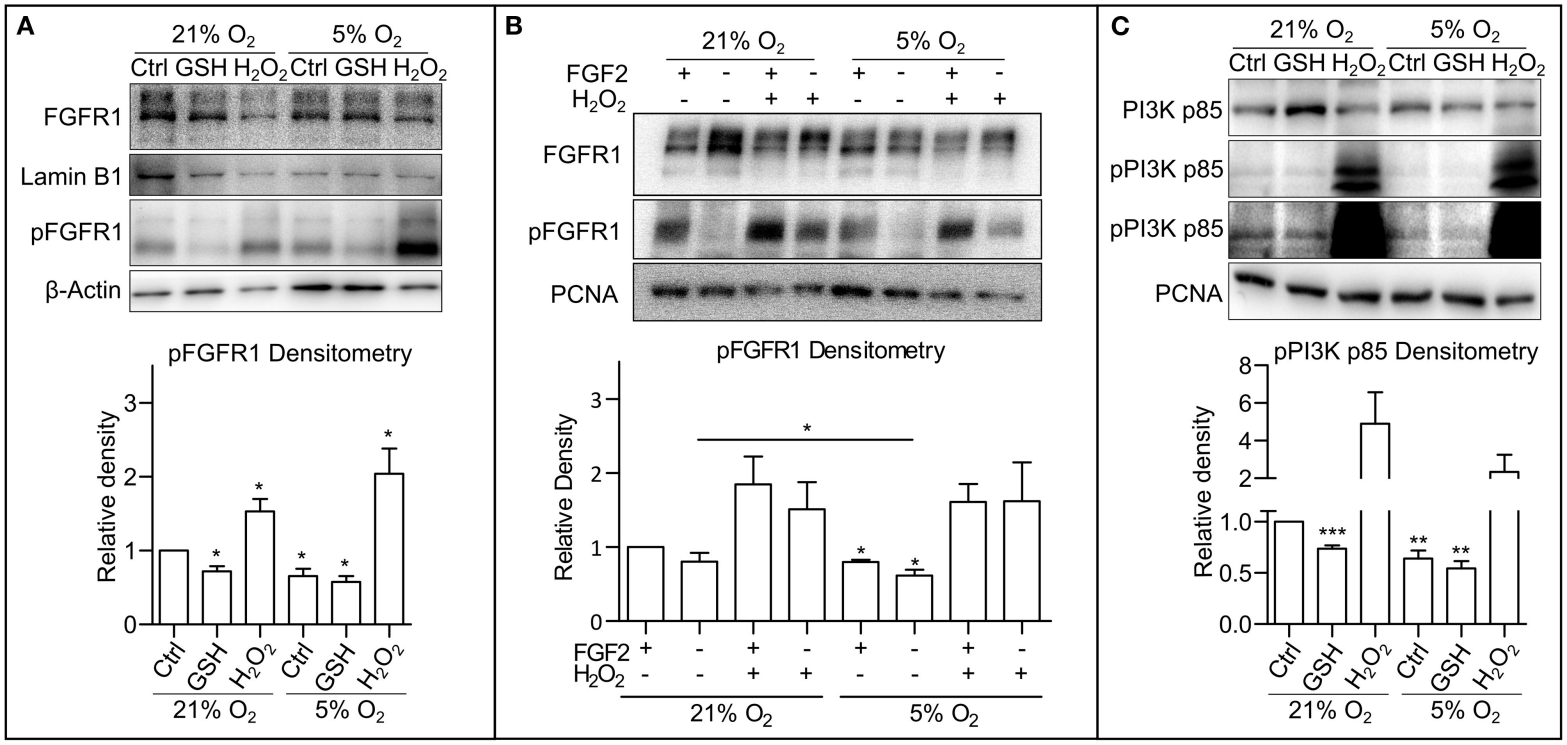
Oxygen-induced ROS upregulate phosphorylation of FGFR1 and PI3K p85. **(A)** Representative WB analysis and quantification of ROS-induced changes in FGFR1 phosphorylation. Cells were cultivated in CM+ for 24 h in 21 and 5% O_2_, respectively, then treated with 10 mM GSH and 0.5 mM H_2_O_2_ for 1 h in fresh media. β-Actin was used as a loading control for pFGFR1 and Lamin B1 for FGFR1. Data obtained on CCTL12, CCTL14, and AM13 hPSCs were used in the densitometry analysis. Data are presented as mean ± SEM (21% O_2_: Ctrl, 1 ± 0, *n* = 7; GSH, 0.7211 ± 0.0690, *n* = 6; H_2_O_2_, 1.530 ± 0.1669, *n* = 5; 5% O_2_: Ctrl, 0.6562 ± 0.0977, *n* = 7; GSH, 0.5748 ± 0.0801, *n* = 5; H_2_O_2_, 2.2038 ± 0.3416, *n* = 5). Statistical analysis was conducted using one sample *t-*test (theoretical mean = 1) with statistical significance taken where *p < 0.05. **(B)** WB analysis of exogenous FGF2-independent ROS-induced FGFR1 phosphorylation. Cells kept in 21 or 5% O_2_ were starved for 24 h and subsequently treated with FGF2 (10 ng/ml) and H_2_O_2_ (1 mM) for 1 h. PCNA was used as a loading control. Data in graph are presented as mean ± SEM (21% O_2_: FGF2/–, 1 ± 0; –/–, 0.803 ± 0.199, *n* = 3; FGF2/H_2_O_2_, 1.845 ± 0.379, *n* = 3; –/H_2_O_2_, 1.511 ± 0.365, *n* = 3; 5% O_2_: FGF2/–, 0.797 ± 0.033, *n* = 3; –/–, 0.617 ± 0.075, *n* = 3; FGF2/H_2_O_2_, 1.608 ± 0.243, *n* = 3; –/H_2_O_2_, 1.621 ± 0.524, *n* = 3). Statistical analysis was conducted using paired one-tailed *t-*test with significance taken where *p < 0.05. **(C)** Representative WB analysis of ROS-induced changes in PI3K p85 phosphorylation in CCTL14 hESCs. Cells were cultivated in CM+ for 24 h in 21 and 5% O_2_, respectively, then treated with 10 mM GSH and 0.5 mM H_2_O_2_ for 1 h in fresh media. PCNA was used as a loading control. Data obtained on CCTL12, CCTL14, and AM13 hPSCs were used in the densitometry analysis. Data are presented as mean ± SEM (21% O_2_: Ctrl, 1 ± 0, *n* = 7; GSH, 0.7335 ± 0.0323, *n* = 6; H_2_O_2_, 4.907 ± 1.657, *n* = 7; 5% O_2_: Ctrl, 0.6401 ± 0.0787, *n* = 6, GSH, 0.5416 ± 0.0731, *n* = 6; H_2_O_2_, 2.335 ± 0.9237, *n* = 5). Statistical analysis was conducted using one sample *t-*test (theoretical mea*n* = 1) with statistical significance taken where **p < 0.01 and ***p < 0.001. Please refer to Raw Images 1 for uncropped WB and to Supplementary Data 1 for source data used to generate the graphs shown in the figure. WB, western blot; hESCs, human embryonic stem cells; ROS, reactive oxygen species; GSH, glutathione; Ctrl, control; hPSCs, human pluripotent stem cells; CM+, conditioned media with FGF2 (10 ng/ml); PCNA, proliferating cell nuclear antigen; FGFR1, fibroblast growth factor receptor 1; pFGFR1, phosphorylated fibroblast growth factor receptor 1; PI3K p85, p85 subunit of phosphatydylinositol-4,5-bisphosphate 3-kinase; pPI3K p85, phosphorylated p85 subunit of phosphatydylinositol-4,5-bisphosphate 3-kinase.

It has been previously described that ROS are able to activate receptor tyrosine kinases (RTKs), either directly causing their autophosphorylation (Chiarugi and Buricchi, 2007) or by inhibition of protein tyrosine phosphatases (Chiarugi and Cirri, 2003; Östman et al., 2011). Thus, to pinpoint the particular mechanism by which ROS modulate PI3K/AKT signaling in hPSCs, we looked upstream of AKT at FGF receptor 1 (FGFR1), the most abundantly expressed FGF receptor in hESCs (Dvorak et al., 2005). Using Western blot, we observed a significantly lower level of FGFR1 Tyr653/654 activating phosphorylation in mild hypoxia compared to 21% O_2_. The treatment with H_2_O_2_ (0.5 mM/1 h) led to upregulation of FGFR1 phosphorylation in both 21 and 5% O_2_, while the GSH treatment (10 mM/1 h) led to a significant downregulation of FGFR1 phosphorylation only in 21% O_2_, in a fashion similar to AKT phosphorylation (Figure 3A). This was also observed in the CCTL12 hESC (Supplementary Figure 3A) and AM13 iPSCs (Supplementary Figure 3B). To elucidate the role of ROS-induced FGFR1 dimerization and autophosphorylation under different oxygen concentrations, we compared FGFR1 phosphorylation with and without exogenous stimulation by FGF2 in 21% O_2_, 5% O_2_, and in H_2_O_2_ (1 mM/1 h)-treated cells. Our results show that H_2_O_2_-mediated ROS induction is sufficient to induce FGFR1 phosphorylation regardless of the exogenous stimulation by FGF2. ROS, therefore, seem to induce dimerization and autophosphorylation of FGFR1 in hPSCs (Figure 3B). Moreover, a stronger FGFR1 phosphorylation was detected in cells in 21% O_2_ compared to 5% O_2_ without FGF2 stimulation (Figure 3B), indicating that the difference in ROS concentration between 21% O_2_ and 5% O_2_ environment (Figure 1C) is sufficient to promote FGFR autophosphorylation independent of exogenous stimuli.

When inhibited, the PI3K's regulatory subunit p55/85 is bound to the p110 catalytic subunit. Upon phosphorylation, p55/85 releases p110, which then catalyzes the conversion of phosphatidylinositol 4,5-diphosphate to phosphatidylinositol 3,4,5-triphosphate (PIP_3_). Figure 3C shows that PI3K p85 phosphorylation was upregulated in 21% O_2_ when compared to mild hypoxia, and H_2_O_2_ led to a massive upregulation of PI3K p85 phosphorylation under both conditions. Treatment with GSH resulted in a significant downregulation of PI3K p85 phosphorylation (Figure 3C) corresponding with the GSH-induced changes in FGFR1 (Figure 3A and Supplementary Figures 3A,B) and AKT phosphorylation (Figure 2C and Supplementary Figures 2A,B). These observations were also made in CCTL12 hESCs (Supplementary Figure 3C) and AM13 iPSCs (Supplementary Figure 3D). Taken together, these findings suggest that ROS upregulate FGFR1 phosphorylation either directly or via inhibition of phosphatases, which subsequently leads to PI3K and AKT phosphorylation.

### ROS Are Attenuated in HIF-1-Dependent Manner

**Figure 4:**
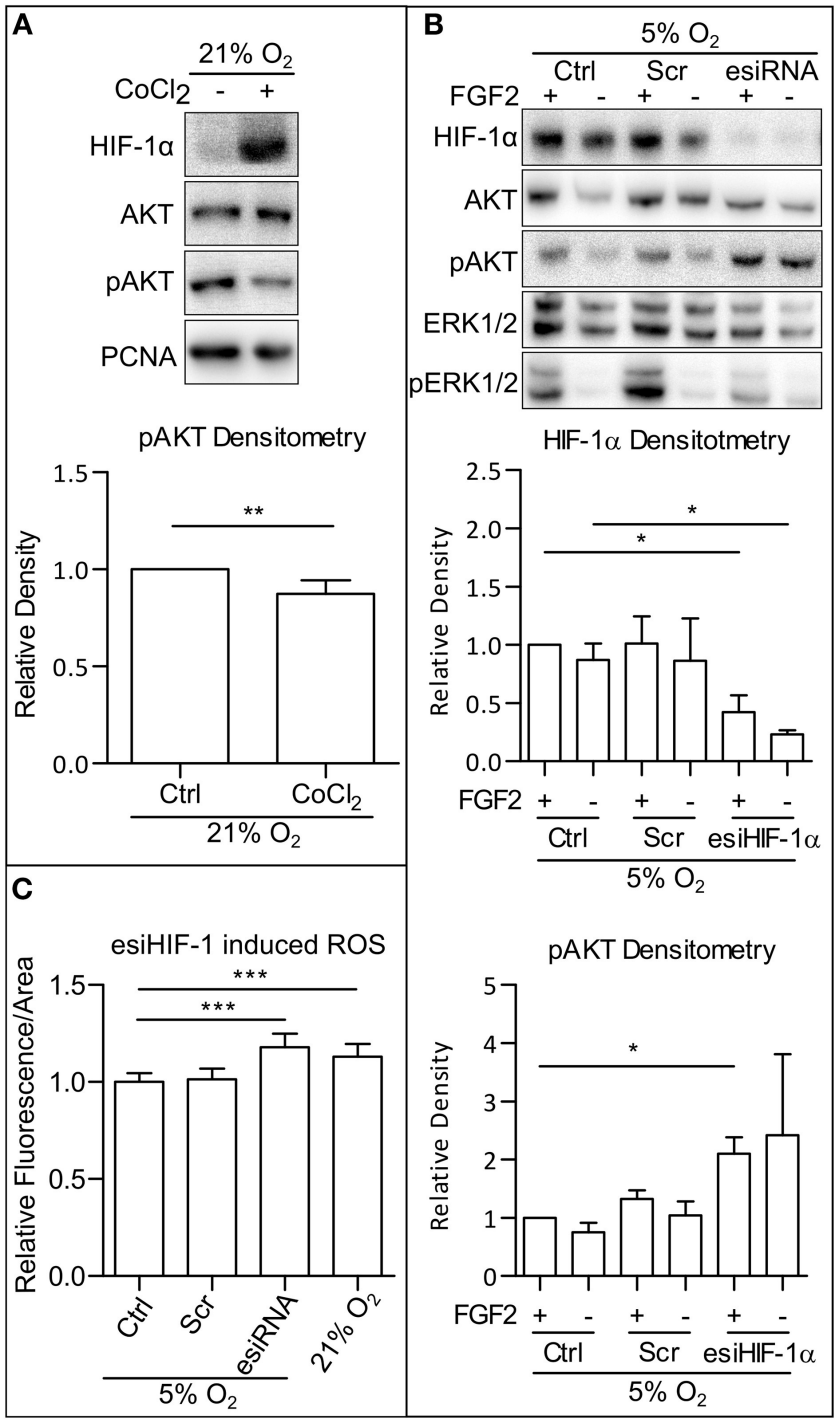
HIF-1 downregulate AKT phosphorylation via downregulation of ROS. **(A)** WB analysis of CoCl_2_-induced HIF-1α stabilization and decrease in AKT phosphorylation in 21% O_2_. Cells were cultivated in CM+ with or without 50 mM CoCl_2_; samples were collected 1 h after fresh media change. PCNA was used as a loading control. Graph represents mean ± SEM (Ctrl, 1 ± 0; CoCl_2_, 0.874 ± 0.028, *n* = 6). Statistical analysis was performed using one samples *t-*test (theoretical mean = 1) with statistical significance taken where **p < 0.01. **(B)** WB analysis of HIF-1α knockdown effect on AKT phosphorylation in 5% O_2_. Cells kept in 21% O_2_ in CM+ were transfected with an esiRNA targeting HIF-1α for 48 h and then transferred to 5% O_2_ and further cultivated with CM- for 24 h. Subsequently, fresh CM– was added, and respective samples were treated with FGF2 (10 ng/ml) for 2 h. Total AKT and ERK1/2 were used as loading controls. Graphs represent mean ± SEM (HIF-1α: Ctrl/+, 1 ± 0; Ctrl/–, 0.87 ± 0.141, *n* = 3; Scr/+, 1.011 ± 0.235, *n* = 4; Scr/–, 0.864 ± 0.362, *n* = 3; esiHIF/+, 0.422 ± 0.143, *n* = 4; esiHIF/–, 0.233 ± 0.034, *n* = 3; pAKT: Ctrl/+, 1 ± 0; Ctrl/–, 0.75 ± 0.162, *n* = 3; Scr/+, 1.324 ± 0.15, *n* = 4; Scr/–, 1.042 ± 0.243, *n* = 3; esiHIF/+, 2.1 ± 0.284, *n* = 4; esiHIF/–, 2.419 ± 1.394, *n* = 3). Statistical analysis was performed using one sample *t-*test (theoretical mean = 1) and paired one-tailed *t-*test with statistical significance taken where *p < 0.05. **(C)** Analysis of HIF-1α knockdown induced ROS in CCTL14 hESCs. Normalized data from three different experiments (with at least 10 individual measurements in each) are expressed as mean ± SD (Ctrl, 1 ± 0.0455, *n* = 36; Scr, 1.013 ± 0.0563, *n* = 43; esiRNA, 1.179 ± 0.0709, *n* = 44; 21% O_2_, 1.131 ± 0.0644, *n* = 42). Statistical significance was calculated using a two-tailed Mann–Whitney test (***p < 0.0001). Please refer to Raw Images 1 for uncropped WB and to Supplementary Data 1 for source data used to generate the graphs shown in the figure. WB, western blot; ROS, reactive oxygen species; GSH, glutathione; Ctrl, control; hPSCs, human pluripotent stem cells; CM+, conditioned media with FGF2 (10 ng/ml); Scr, scrambled short-interfering RNA; esiRNA, endoribonuclease-prepared short-interfering RNA; AKT, protein kinase B; pAKT, phosphorylated protein kinase B; ERK1/2, extracellular signal-regulated kinase 1 and 2; pERK1/2, phosphorylated extracellular signal-regulated kinase 1 and 2; PCNA, proliferating cell nuclear antigen; HIF-1α, hypoxia-inducible factor 1α.

To understand what contributes to the ROS downregulation in mild hypoxia, we looked at the effect of HIFs, the main facilitators of cellular adaptation to hypoxic conditions (Semenza, 2001; Keith et al., 2012). First, we employed CoCl_2_ (50 mM), a hypoxia mimetic capable of stabilizing the alpha subunits of HIFs in 21% O_2_ (Figure 4A). In our cells, CoCl_2_ treatment led to a decrease in AKT phosphorylation, comparable to the decrease observed in mild hypoxia (Figures 1A,B and Supplementary Figures 1A,B). It has been previously established that CoCl_2_ stabilizes alpha subunits of all HIFs, but only HIF-1α is transcriptionally active under CoCl_2_ treatment (Befani et al., 2013). Next, we silenced HIF-1α expression using endoribonuclease-prepared short-interfering RNA (esiRNA). This caused a decrease in ERK1/2 phosphorylation and an increase in AKT phosphorylation independent of FGF2 presence (Figure 4B), which was also observed in AM13 iPSCs (Supplementary Figure 4A). To determine if HIF-1α-mediated phospho-AKT downregulation is also associated with the changing levels of ROS, we compared ROS levels in cells maintained in 5% O_2_, in hESCs treated and untreated with esiRNA targeting HIF-1α, and in cells grown in 21% O_2_. The silencing of HIF-1α expression induced a significant increase in ROS in 5% O_2_, an amount comparable to that observed in 21% O_2_ (Figure 4C). These results suggest that the upregulation of AKT phosphorylation observed in hPSCs following HIF-1α silencing is associated with ROS upregulation.

### MAPK Downregulate PI3K/AKT via ROS Attenuation

**Figure 5:**
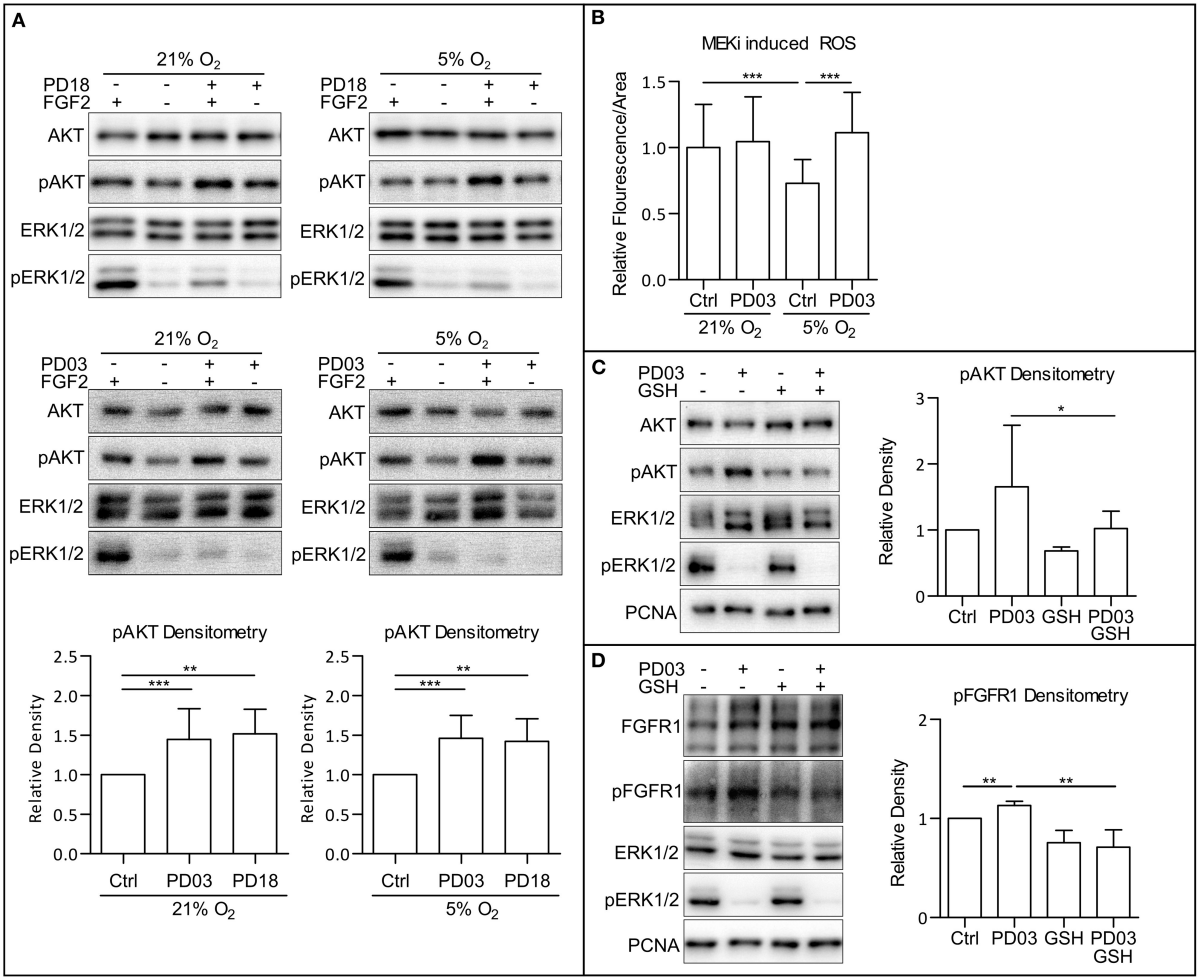
MAPK pathway downregulates PI3K/AKT via downregulation of ROS. **(A)** WB analysis of MEK1/2 inhibition-induced upregulation of AKT phosphorylation. Cells were cultivated in 21 and 5% O_2_ in CM– 24 h prior the experiment; then, respective cells were treated with FGF2 (10 ng/ml), PD184352 (PD18, 25 μM), and PD0325901 (PD03, 0.2 μM) for 2 h in respective oxygen concentrations. Total AKT and ERK1/2 were used as loading controls. Graphs represent mean ± SD (21% O_2_: Ctrl, 1 ± 0; PD03, 1.447 ± 0.386, *n* = 18; PD18, 1.517 ± 0.312, *n* = 6; 5% O_2_: Ctrl, 1 ± 0; PD03, 1.459 ± 0.292, *n* = 16; PD18, 1.422 ± 0.285, *n* = 9). Statistical significance was calculated using one sample *t-*test (theoretical mea*n* = 1) with statistical significance taken where **p < 0.01 and ***p < 0.001. **(B)** MEK1/2 inhibition upregulates ROS in 5% O_2_ in CCTL14 hESCs. Cells were cultivated in CM+ in respective oxygen concentrations for 24 h and additional 2 h after media change and treatment with PD03 (0.2 μM) the next day. Normalized data from three different experiments (with at least 10 measurements in each) are presented as mean ± SD (21% O_2_, 1 ± 0.3268, *n* = 36; 21% O_2_ PD03, 1.045 ± 0.3406, *n* = 37; 5% O_2_, 0.7304 ± 0.1785, *n* = 36; 5% O_2_ PD03, 1.113 ± 0.3059, *n* = 41). Statistical significance was calculated using a two-tailed Mann–Whitney test (***p < 0.0001). **(C)** GSH reverts MEK1/2-inhibition induced phosphorylation of AKT. Cells were cultivated in 21% O_2_ in CM+. Respective samples were treated with GSH (10 mM) and PD03 (0.2 μM) for 1 h. PCNA was used as a loading control. Graph represents mean ± SD (Ctrl, 1 ± 0; PD03, 1.654 ± 0.933, *n* = 6; GSH, 0.686 ± 0.139, *n* = 6; PD03/GSH, 0.954 ± 0.29, *n* = 6). Statistical significance was calculated using one sample *t-*test (theoretical mea*n* = 1) with statistical significance taken where *p < 0.05. **(D)** GSH reverts MEK1/2-inhibition induced phosphorylation of FGFR1. Cells were cultivated in 21% O_2_ in CM+. Respective samples were treated with GSH (10 mM) and PD03 (0.2 μM) for 1 h. PCNA was used as a loading control. Graph represents mean ± SD (Ctrl, 1 ± 0; PD03, 1.131 ± 0.042, *n* = 5; GSH, 0.754 ± 0.125, *n* = 5; PD03/GSH, 0.709 ± 0.178, *n* = 5). Statistical significance was calculated using one sample *t-*test (theoretical mea*n* = 1) and paired two-tailed *t-*test with statistical significance taken where **p < 0.05. Please refer to Raw Images 1 for uncropped WB and to Supplementary Data 1 for source data used to generate the graphs shown in the figure. WB, western blot; ROS, reactive oxygen species; GSH, glutathione; Ctrl, control; CM+, conditioned media with FGF2 (10 ng/ml); CM–, conditioned media without FGF2; PD18, PD184161; PD03, PD0325901; AKT, protein kinase B; pAKT, phosphorylated protein kinase B; ERK1/2, extracellular signal-regulated kinase 1 and 2; pERK1/2, phosphorylated extracellular signal-regulated kinase 1 and 2; MEK1/2, mitogen activated protein kinase kinase 1 and 2; PCNA, proliferating cell nuclear antigen; FGFR1, fibroblast growth factor receptor 1; pFGFR1, phosphorylated fibroblast growth factor receptor 1.

Since we observed a simultaneous increase in AKT phosphorylation and a decrease in ERK1/2 phosphorylation following the HIF-1α knockdown by esiRNA, we wondered whether MAPK could play a role in PI3K/AKT regulation in hPSCs. Crosstalk between the MAPK and PI3K/AKT pathway was previously described (Aksamitiene et al., 2012). In order to analyze the effect of MAPK on PI3K/AKT, we used two small molecule inhibitors of mitogen-activated protein kinase kinase 1 and 2 (MEK1/2), PD184161 (PD18), and PD0325901 (PD03) to treat cells cultivated either in 21% O_2_ or 5% O_2_. Comparing the results, it can be seen that the downregulation of ERK1/2 phosphorylation leads to the upregulation of AKT phosphorylation in 21% O_2_ in the presence of FGF2 in CCTL14 and CCTL12 hESCs and AM13 iPSCs (Figure 5A and Supplementary Figures 5A,B). These results suggest a negative impact of FGF2-induced MAPK signaling on PI3K/AKT pathway activation in hPSCs.

To assess whether MAPK signaling modulates ROS, we analyzed ROS levels in hESC line CCTL14 treated with a MEK1/2 inhibitor PD03 under both 21 and 5% O_2_. MEK1/2 inhibition leads to a statistically insignificant (*p* = 0.145) upregulation of ROS in 21% O_2_ and a significant upregulation of ROS in 5% O_2_ (Figure 5B) compared to untreated cells maintained in corresponding O_2_ concentrations. Considering that both the maintenance of cells in 21% O_2_ and MEK1/2 inhibition in mild hypoxia were sufficient to raise ROS and elevated ROS are associated with increased AKT phosphorylation, it is possible that MAPK suppresses PI3K/AKT signaling via modulation of ROS levels as second messengers.

We, therefore, combined the ROS scavenger GSH (10 mM) and MEK1/2 inhibitor PD03 in 21% O_2_. This led to a rescue of the PD03-induced AKT phosphorylation (Figure 5C), implicating that MAPK downregulates AKT phosphorylation via modulation of ROS. Same results were acquired in CCTL12 hESCs (Supplementary Figure 5C) and AM13 iPSCs (Supplementary Figure 5D). To further validate this hypothesis, we also looked at FGFR1 Tyr653/654-activating phosphorylation, which we previously detected to be upregulated by ROS (Figures 3A,B and Supplementary Figures 3C,D). Our results show that FGFR1 phosphorylation is also upregulated by MEK1/2 inhibition, and the addition of GSH rescues this effect in 21% O_2_ in all three hPSCs lines used in this study (Figure 5D and Supplementary Figures 5C,E). In summary, our results suggest that MAPK can downregulate PI3K/AKT phosphorylation via the downregulation of second messengers—intracellular ROS.

### ROS Downregulate PI3K/AKT via PTEN Inhibition

**Figure 6:**
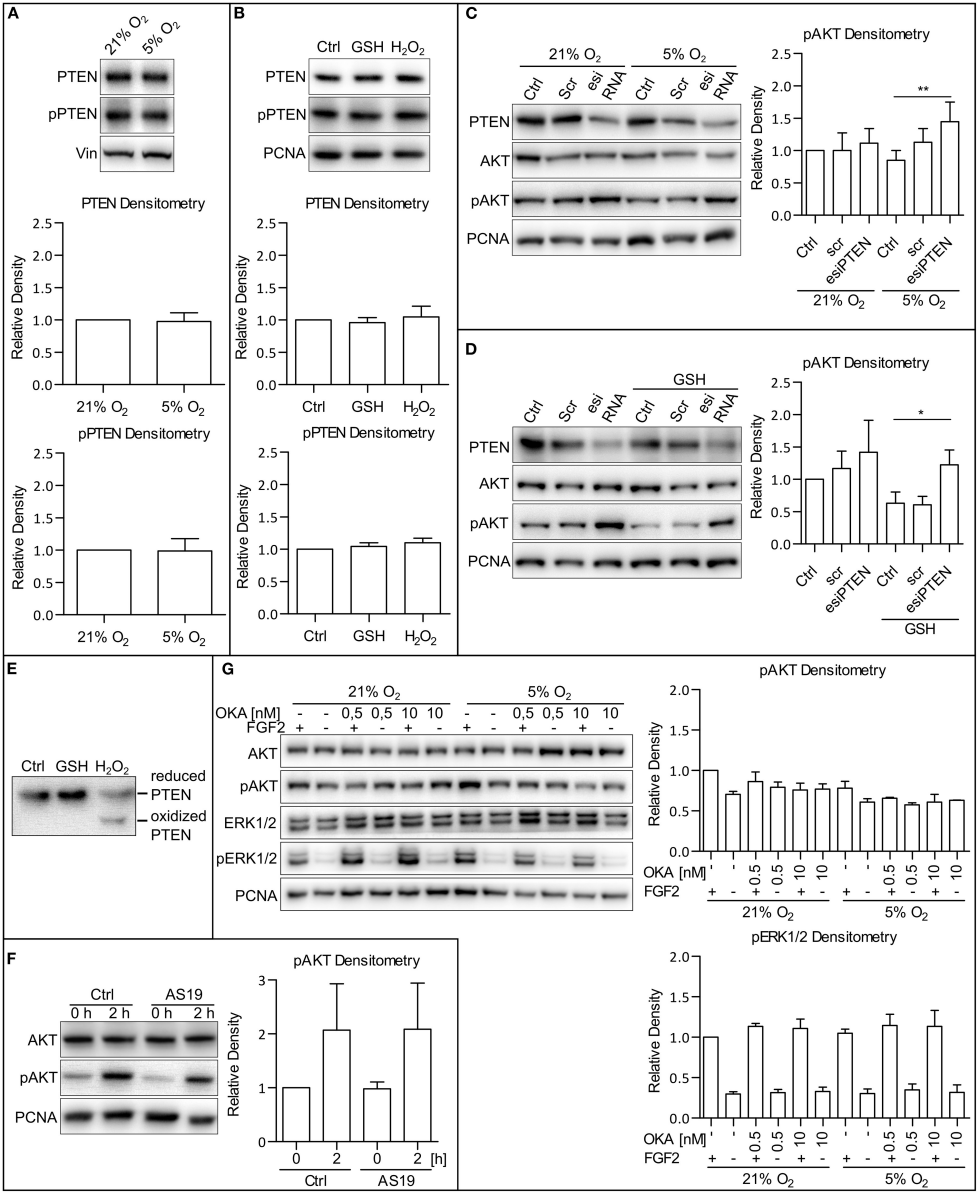
PTEN activity is downregulated by ROS. **(A)** WB analysis of PTEN expression and phosphorylation dependent on oxygen concentration. Cells were cultivated in CM+ and respective oxygen concentrations for 24 h and additional 2 h after fresh CM+ change. Vinculin (Vin) was used as a loading control. Graphs represent mean ± SD (PTEN: 21% O_2_, 1 ± 0; 5% O_2_, 0.977 ± 0.134, *n* = 10; pPTEN: 21% O_2_, 1 ± 0; 5% O_2_, 0.988 ± 0.189, *n* = 10). Statistical significance was calculated using one sample *t-*test (theoretical mea*n* = 1). **(B)** WB analysis of the impact of ROS on PTEN expression and phosphorylation. Cells were cultivated in CM+ for 24 h and an additional hour after fresh CM+ change and GSH (10 mM) and H_2_O_2_ (0.5 mM) treatment. PCNA was used as a loading control. Graphs represent mean ± SEM (PTEN: Ctrl, 1 ± 0; GSH, 0.958 ± 0.078, *n* = 4; H_2_O_2_, 1.046 ± 0.167, *n* = 4; pPTEN: Ctrl, 1 ± 0; GSH, 1.042 ± 0.058, *n* = 4; H_2_O_2_, 1.099 ± 0.071, *n* = 4). Statistical significance was calculated using one sample *t-*test (theoretical mea*n* = 1). **(C)** The silencing of PTEN expression upregulates AKT phosphorylation more substantially in 5% compared to the 21% O_2_ environment. Cells cultivated in 21% O_2_ in CM+ were transfected with an esiRNA targeting PTEN for 48 h, and respective cells were transferred to 5% O_2_ 24 h before the harvesting. PCNA was used as a loading control. Graph represents mean ± SD (21% O_2_: Ctrl, 1 ± 0; Scr, 1 ± 0.274, *n* = 6; esiPTEN, 1.116 ± 0.225, *n* = 6; 5% O_2_: Ctrl, 0.851 ± 0.15, *n* = 6; Scr, 1.129 ± 0.211, *n* = 6; esiPTEN, 1.445 ± 0.305, *n* = 6). Statistical significance was calculated using paired two-tailed *t-*test with significance taken where **p < 0.01. **(D)** AKT phosphorylation after PTEN silencing is more pronounced in GSH-treated cells compared to the control in CCTL14 hESCs. Cells cultivated in 21% O_2_ in CM+ were transfected with esiRNA targeting PTEN. Respective cells were treated with GSH (10 mM) 1 h prior to harvesting. PCNA was used as a loading control. Graph represents mean ± SD (21% O_2_: Ctrl, 1 ± 0; Scr, 1.166 ± 0.268, *n* = 5; esiPTEN, 1.416 ± 0.495, *n* = 5; GSH: Ctrl, 0.63 ± 0.172, *n* = 5; Scr, 0.605 ± 0.131, *n* = 5; esiPTEN, 1.225 ± 0.229, *n* = 5). Statistical significance was calculated using paired two-tailed *t-*test with significance taken where *p < 0.05. **(E)** WB analysis of PTEN oxidation in hPSCs. Cells were treated with 10 mM GSH and 0.5 mM H_2_O_2_ for 1 h and harvested in native lysis buffer. Oxidized PTEN migrated faster on 8% gel due to H_2_O_2_-induced disulfides. **(F)** WB analysis of cells treated with SHIP2 inhibitor. Cells were cultivated in CM+ in 21% O_2_. Twenty-four hours prior to the treatment, CM– was administered, and the cells were transferred to 5% O_2_. The next day, fresh CM– was administered, and the cells were treated with FGF2 (10 ng/ml) and AS1938909 (AS19, 10 μM) for 0 or 2 h. PCNA was used as a loading control. Graph represents mean ± SD (21% O_2_: 0 h, 1 ± 0; 2 h, 2.069 ± 0.86, *n* = 4; 5% O_2_: 0 h, 0.979 ± 0.314, *n* = 6; 2 h, 2.089 ± 0.853, *n* = 4). Statistical significance was calculated using one sample *t-*test (theoretical mea*n* = 1). **(G)** WB analysis of PP2A and PP1 inhibition with okadaic acid in CCTL14 hESCs. Cells were cultivated in CM+ in 21% O_2_ for 24 h; then, CM– was administered, and the cells were transferred to 5% O_2_. The following day, fresh CM– was administered once again, and the cells were treated for 2 h with FGF2 (10 ng/ml) and okadaic acid (OKA) in 0.5 and 10 nM concentrations, specific for PP2A and PP1 inhibition, respectively. PCNA was used as a loading control. Graphs represent mean ± SEM (pAKT: 21% O_2_: FGF2/–, 1 ± 0; –/–, 0.704 ± 0.037, *n* = 3; FGF2/0.5, 0.862 ± 0.120, *n* = 3; –/0.5, 0.793 ± 0.064, *n* = 3; FGF2/10, 0.759 ± 0.083, *n* = 3; –/10, 0.77 ± 0.064; 5% O_2_: FGF2/–, 0.781 ± 0.086, *n* = 3; –/–, 0.61 ± 0.039, *n* = 3; FGF2/0.5, 0.656 ± 0.12, *n* = 3; –/0.5, 0.576 ± 0.024, *n* = 3; FGF2/10, 0.609 ± 0.96, *n* = 3; –/10, 0.633 ± 0.001; pERK1/2: 21% O_2_: FGF2/–, 1 ± 0; –/– 0.299 ± 0.028, *n* = 3; FGF2/0.5, 1.136 ± 0.034, *n* = 3; –/0.5, 0.315 ± 0.039, *n* = 3; FGF2/10, 1.109 ± 0.116, *n* = 3; –/10, 0.328 ± 0.053; 5% O_2_: FGF2/–, 1.05 ± 0.051, *n* = 3; –/–, 0.304 ± 0.056, *n* = 3; FGF2/0.5, 1.146 ± 0.138, *n* = 3; –/0.5 0.348 ± 0.075, *n* = 3; FGF2/10, 1.134 ± 0.198, *n* = 3; –/10, 0.316 ± 0.094). Statistical significance was calculated using one sample *t-*test (theoretical mean = 1). Please refer to Raw Images 1 for uncropped WB and to Supplementary Data 1 for source data used to generate the graphs shown in the figure. WB, western blot; ROS, reactive oxygen species; GSH, glutathione; Ctrl, control; CM+, conditioned media with FGF2 (10 ng/ml); scr, scrambled short interfering RNA; esiRNA, endoribonuclease-prepared short interfering RNA; AKT, protein kinase B; pAKT, phosphorylated protein kinase B; ERK1/2, extracellular signal-regulated kinase 1 and 2; pERK1/2, phosphorylated extracellular signal-regulated kinase 1 and 2; PCNA, proliferating cell nuclear antigen; PTEN, phosphatase and tensin homolog deleted on chromosome 10; pPTEN, phosphorylated phosphatase and tensin homolog deleted on chromosome 10; Vin, Vinculin; PP1, protein phosphatase 1; PP2A, protein phosphatase 2A; AS19, AS1938909; OKA, okadaic acid.

To establish the molecular mechanism by which MAPK and oxygen-induced ROS modulation leads to the AKT phosphorylation changes, we analyzed a set of candidate phosphatases possibly involved in the regulation of AKT phosphorylation: the phosphatase and tensin homolog protein deleted on chromosome 10 (PTEN), SH2-domain containing inositol-5′-phosphatase 2 (SHIP2), and protein phosphatase 2A (PP2A). PTEN, a primary antagonist of PI3K, has been described to be regulated in several ways: by its total amount, its phosphorylation (Vazquez et al., 2001), or by the formation of reversible ROS-induced disulfide bridges (Lee et al., 2002; Covey et al., 2007; Kim et al., 2018). We observed that the total amount of PTEN, as well as the extent of its phosphorylation, did not differ in 5 and 21% O_2_ in CCTL14 and CCTL12 hESCs and AM13 iPSCs (Figure 6A and Supplementary Figures 6A,B). We also did not observe any changes in the total amount of PTEN and PTEN phosphorylation after direct modulation of ROS levels by H_2_O_2_ (0.5 mM/1 h) and GSH (10 mM/1 h) in these cell lines (Figure 6B and Supplementary Figures 6C,D). Interestingly, after silencing PTEN expression by esiRNA and comparing the levels of phosphorylated AKT, we noted a slight increase in AKT phosphorylation in 21% O_2_ and a significant upregulation in 5% O_2_ in all three hPSCs lines used in this study (Figure 6C and Supplementary Figures 6E,G). Moreover, when we combined PTEN silencing with a GSH treatment to induce ROS downregulation in 21% O_2_, AKT phosphorylation appeared to be significantly elevated in GSH-treated hPSCs with downregulated PTEN compared to GSH-free cells (Figure 6D and Supplementary Figures 6F,G). To analyze whether PTEN can undergo oxidization in hPSCs, we collected native lysates of H_2_O_2_-treated and control cells and performed 8% SDS-PAGE to detect an oxidation-induced shift in PTEN mobility (Lee et al., 2002; Covey et al., 2007). The results show that ROS-induced PTEN oxidation takes place in hPSCs as well (Figure 6E). Overall, these data indicate that PTEN is more active in a low ROS environment, which could partially contribute to lower levels of AKT phosphorylation observed in mild hypoxia. Although we did not detect oxygen or ROS-mediated changes in PTEN total amount or its phosphorylation, our data suggest that it is the ROS-regulated PTEN activity that contributes to the ROS-mediated upregulation of AKT phosphorylation.

### SHIP2 and PP2A Are Not Involved in the Immediate Mild Hypoxia-Induced Downregulation of AKT Phosphorylation

SHIP2 is an antagonist of PI3K functionally similar to PTEN that is described to be regulated by ROS (Zhang et al., 2007). To analyze whether SHIP2 facilitates the downregulation of AKT phosphorylation in mild hypoxia, we treated hPSCs in 5% O_2_ with a specific SHIP2 inhibitor AS1938909 (AS19, 10 μM/2 h). hPSCs treated with AS19 did not display a significantly elevated AKT phosphorylation than untreated control (Figure 6F and Supplementary Figures 7A,B), suggesting that SHIP2 is not involved in the observed downregulation of PI3K/AKT in mild hypoxia.

PP2A directly regulates AKT and ERK1/2 phosphorylation and is also described to be regulated by ROS (Rao and Clayton, 2002; Raman and Pervaiz, 2019). To analyze its role in the regulation of AKT in hPSCs, we used a 2-h okadaic acid (OKA) treatment in the 0.5 nM concentration specific for PP2A and in the 10 nM concentration specific for protein phosphatase 1 (PP1). Treatment with OKA in both concentrations did not significantly enhance AKT or ERK1/2 phosphorylation in hPSCs regardless of O_2_ concentration (Figure 6G and Supplementary Figures 7C,D). This, together with the fact that PP2A targets both AKT and ERK1/2 and that we did not see ERK1/2 downregulation in mild hypoxia, suggests that it has no role in the observed hypoxia-mediated downregulation of AKT phosphorylation in hPSCs.

### ROS Attenuation Upregulates Pluripotency Markers

**Figure 7:**
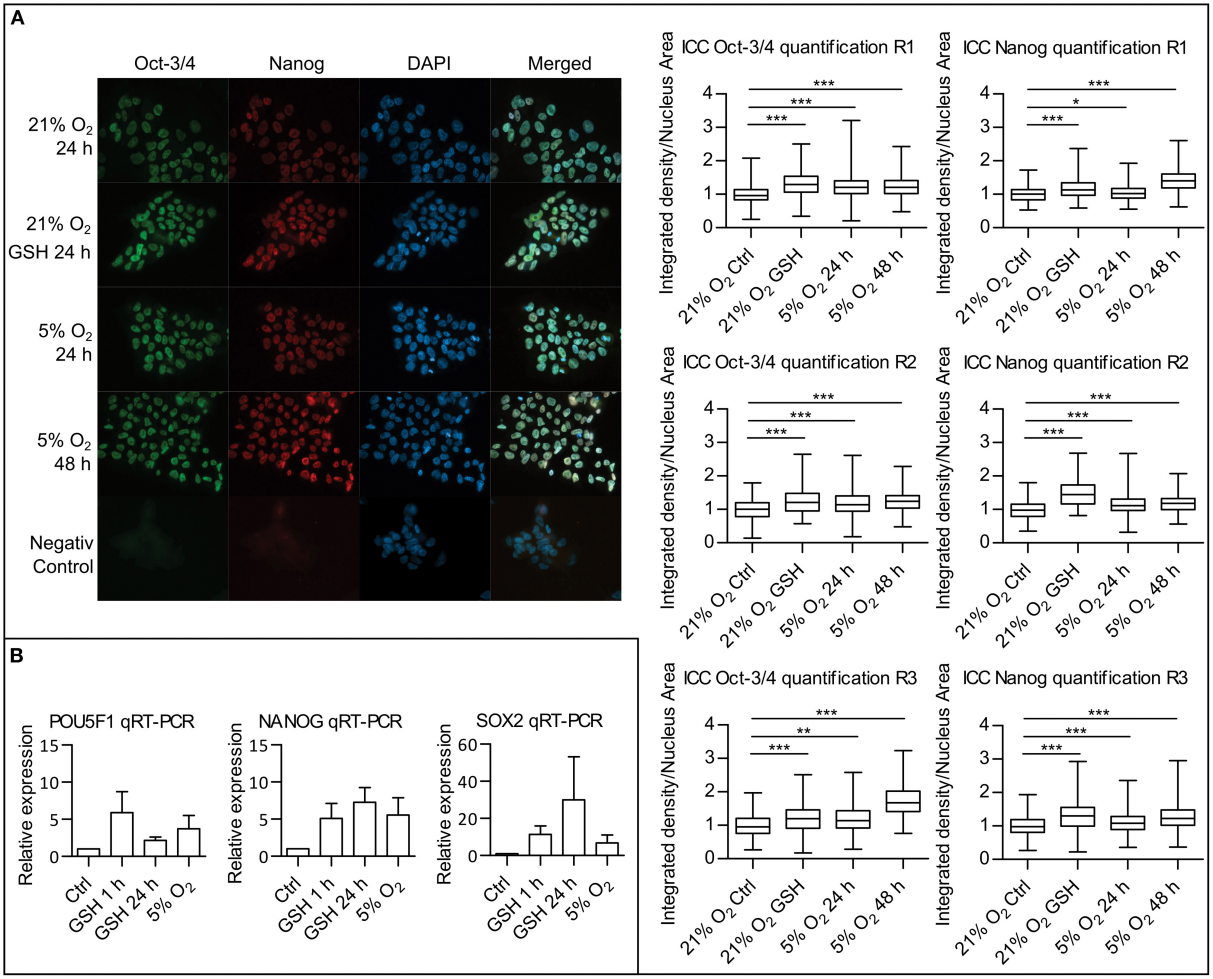
ROS scavenging by GSH increases pluripotency markers expression. **(A)** ICC analysis of Oct-3/4 and Nanog proteins amounts with quantification. hPSCs in 21% O_2_ with and without GSH (10 mM) were stained and fixed after 24 h together with hPSCs, which were in 5% O_2_ for 24 and 48 h. Representative snapshots are presented. Graphs on the right show relative quantification of signal density per nuclear area for individual nuclei from three repetitions (R1–3). Box plots show mean, 25–75% percentile range, and whiskers show minimal and maximal value range. Mean values and standard deviations for Oct-3/4: R1: 21% O_2_, 1 ± 0.241, *n* = 364; 21% O_2_ GSH, 1.306 ± 0.332, *n* = 397; 5% O_2_ 24 h, 1.235 ± 0.317, *n* = 465; 5% O_2_ 48 h, 1.225 ± 0.295, *n* = 647; R2: 21% O_2_, 1 ± 0.280, *n* = 230; 21% O_2_ GSH, 1.240 ± 0.408, *n* = 68; 5% O_2_ 24 h, 1.202 ± 0.407, *n* = 231; 5% O_2_ 48 h, 1.249 ± 0.316, *n* = 230; R3: 21% O_2_, 1 ± 0.311, *n* = 267; 21% O_2_ GSH, 1.197 ± 0.423, *n* = 135; 5% O_2_ 24 h, 1.197 ± 0.396, *n* = 231; 5% O_2_ 48 h, 1.715 ± 0.436, *n* = 356. Mean values and standard deviations for Nanog: R1: 21% O_2_, 1 ± 0.223, *n* = 376; 21% O_2_ GSH, 1.173 ± 0.280, *n* = 392; 5% O_2_ 24 h, 1.038 ± 0.217, *n* = 463; 5% O_2_ 48 h, 1.421 ± 0.323, *n* = 645; R2: 21% O_2_, 1 ± 0.264, *n* = 249; 21% O_2_ GSH, 1.465 ± 0.376, *n* = 69; 5% O_2_ 24 h, 1.144 ± 0.303, *n* = 232; 5% O_2_ 48 h, 1.18 ± 0.261, *n* = 217; R3: 21% O_2_, 1 ± 0.299, *n* = 279; 21% O_2_ GSH, 1.309 ± 0.421, *n* = 132; 5% O_2_ 24 h, 1.086 ± 0.305, *n* = 251; 5% O_2_ 48 h, 1.268 ± 0.339, *n* = 309. Statistical significance was calculated using unpaired two-tailed *t-*test with significance taken where *p < 0.05, **p < 0.01, and ***p < 0.0001. **(B)** Quantitative real-time PCR analysis of pluripotency genes POU5F1, NANOG, and SOX2 expression. Graphs show relative expression of pluripotency genes in untreated hPSCs controls, hPSCs treated with GSH (10 mM) for 1 and 24 h, and hPSCs maintained in 5% O_2_ for 24 h. Graphs represent mean ± SEM (POU5F1: Ctrl, 1 ± 0; GSH 1 h, 5.880 ± 2.834, *n* = 4; GSH 24 h, 2.158 ± 0.453, *n* = 4; 5% O_2_, 3.740 ± 1.770, *n* = 4; NANOG: Ctrl, 1 ± 0; GSH 1 h, 5.110 ± 2.008, *n* = 5; GSH 24 h, 7.260 ± 1.983, *n* = 4; 5% O_2_, 5.572 ± 2.322, *n* = 5; SOX2: Ctrl, 1 ± 0; GSH 1 h, 11.38 ± 4.618, *n* = 4; GSH 24 h, 29.98 ± 23.30, *n* = 4; 5% O_2_, 6.848 ± 4.147, *n* = 4). GSH, reduced glutathione; hPSCs, human pluripotent stem cells; ICC, immunocytochemistry; R1–3, repetitions 1–3; ROS, reactive oxygen species; SEM, standard error of mean.

Since we were able to describe a link between attenuated ROS levels in mild hypoxia and a decrease in PI3K/AKT signaling, we proceeded to study the effect of ROS levels on pluripotency maintenance. Using immunocytochemistry (ICC), we compared the amount of two well-established pluripotency markers Oct-3/4 and Nanog in hPSCs in 21% O_2_, in hPSCs with ROS scavenged by GSH (10 mM/24 h), and in hPSCs in 5% O_2_. It has been shown that the effect of hypoxia on the levels of pluripotency markers requires longer incubation times (Forristal et al., 2010; Mathieu et al., 2014). We, therefore, analyzed the Oct-3/4 and Nanog levels after 24 and 48 h in the 5% O_2_ environment. Upon nuclear signal quantification, we observed a significant increase in the nuclear Oct-3/4 and Nanog signal in 5% O_2_ after 24 and 48 h as well as in the GSH-treated cells compared to control in 21% O_2_ (Figure 7A). To distinguish between the amount of protein and changes in the pluripotency markers' gene expression, we also performed quantitative real-time PCR (qRT-PCR) of NANOG, POU5F1, and SOX2 comparing cells cultivated in 21 and 5% O_2_ and cells with ROS scavenged by GSH (10 mM) in 21% O_2_ (Figure 7B). We observed an increase in NANOG, POU5F1, and SOX2 expression following the GSH treatment comparable to levels of expression seen in cells grown in 5% O_2_. qRT-PCR results correlate with the observed significant changes in protein amount, emphasizing the importance of ROS signaling in pluripotency maintenance.

## Discussion

hPSCs are routinely cultivated in media supplemented with FGF2 to support their undifferentiated growth (Dvorak et al., 2005; Xu et al., 2005a; Levenstein et al., 2006; Eiselleova et al., 2009) in atmospheric oxygen pressure (21% O_2_). FGF2 promotes pluripotency and self-renewal through activation of PI3K/AKT and MAPK pathways (Armstrong et al., 2006; Li et al., 2007; Yu et al., 2011; Singh et al., 2012; Hossini et al., 2016; Wang et al., 2017; Haghighi et al., 2018). Previous studies have shown an enhancement of proliferation, transcription of pluripotency markers, generation of iPSCs, and inhibition of differentiation in low oxygen tension (Ezashi et al., 2005; Covello et al., 2006; Yoshida et al., 2009; Forristal et al., 2010; Mathieu et al., 2014), characteristic for the blastocyst's native niche (Fischer and Bavister, 1993; Okazaki and Maltepe, 2006). Hypoxia was described to induce or mediate the upregulation of PI3K/AKT (Alvarez-Tejado et al., 2001; Lee et al., 2013) and MAPK (Miyamoto et al., 2015) pathways in various cell lines. Surprisingly, a recent study conducted on mouse embryonic stem cells (mESCs) showed that PI3K/AKT and MAPK pathways are downregulated in hypoxia (Kučera et al., 2017) despite the fact that PI3K/AKT and MAPK pathways are considered to play an essential role in pluripotency maintenance. In light of this observation, it was of utmost importance to dissect mechanisms employed in hPSCs.

### ROS Upregulate AKT Phosphorylation in hPSCs and Are Downregulated in Mild Hypoxia

We show that the 5% O_2_ environment (referred to as mild hypoxia for the purpose of this study) stabilizes HIF-1α and downregulates AKT phosphorylation in hPSCs, while it does not have a significant effect on MEK1/2–ERK1/2 pathway (Figures 1A,B and Supplementary Figures 1A,B). Hypoxia was previously associated with decreased ROS levels (Kučera et al., 2017), and ROS were shown to regulate various cellular signaling pathways (Rhee, 2006; Genestra, 2007; Zhang et al., 2016). ROS were even found to be upregulating the MAPK and PI3K/AKT signaling in mESCs where hypoxia was associated with downregulation of these pathways (Kučera et al., 2017).

Decreased ROS levels are associated with mild hypoxia in hESCs as well (Figure 1C). Their selective downregulation by GSH (Figure 2A) led to PI3K/AKT downregulation independent of oxygen status (Figure 2C and Supplementary Figures 2A,B). Vice versa, selective upregulation of ROS by H_2_O_2_ (Figure 2B) independent of oxygen concentration led to PI3K/AKT and MAPK upregulation (Figure 2C and Supplementary Figures 2A,B). Considering that ROS levels directly respond to oxygen concentration (Figure 1B), ROS seem to act as second messengers responsible for PI3K/AKT upregulation in response to oxygen level. H_2_O_2_ also upregulated ERK1/2 phosphorylation in hPSCs, but ERK1/2 phosphorylation changed neither upon O_2_ concentration changes (Figure 1A and Supplementary Figures 1A,B) nor in the presence of GSH in hPSCs (Figure 2C and Supplementary Figures 2A,B) contrary to mESCs (Kučera et al., 2017). A possible explanation is that MAPK serves different roles in mESCs and hESCs. Upregulation of MAPK in mESCs leads to lineage commitment (Kunath et al., 2007), but self-renewal of hPSCs relies on highly active MAPK (Dvorak et al., 2005; Eiselleova et al., 2009). Thus, high MAPK activity upon FGF2 induction possibly makes hPSCs less sensitive to subtle changes in ROS induced by O_2_ concentration changes. Above that, our data also indicate that the MEK1/2–ERK1/2 pathway attenuates ROS (Figure 5B); the existence of a positive feedback loop from ROS toward mitogenic pathways is, therefore, possible, perhaps on the level of the FGF2 receptor.

### O_2_ Activates FGFR1 and PI3K via ROS in hPSCs

ROS have been described to activate receptor tyrosine kinases (RTKs) through dimerization induced by the oxidation-mediated formation of disulfide bonds between the cysteines of neighboring monomers (Chiarugi and Buricchi, 2007) or by inhibition of protein tyrosine phosphatases via cysteine oxidation (Chiarugi and Cirri, 2003; Chiarugi and Buricchi, 2007). Indeed, phosphorylation of FGFR1, the most abundantly expressed FGFR in hESCs (Dvorak et al., 2005), was upregulated by both ROS and O_2_ in hPSCs (Figures 3A,B and Supplementary Figures 3A,B), suggesting this may contribute to the ROS-mediated upregulation of AKT phosphorylation. The upregulation of FGFR1 phosphorylation by H_2_O_2_ and O_2_ was observed without the exogenous FGF2 stimulation as well (Figure 3B), implying that oxidation mediates the FGFR1 phosphorylation either via promoting its dimerization and autophosphorylation or via inhibition of FGFR1-associated phosphatases.

We also observed ROS-induced phosphorylation of the PI3K regulatory subunit p85, which is necessary for the release and activation of the catalytic p110 subunit. Possibly, this is a result of upstream ROS-mediated FGFR1 phosphorylation. Nevertheless, the lack of O_2_-induced FGFR1–MEK1/2–ERK1/2 response denotes that ROS could directly modulate PI3K p85 in hPSCs (Figure 3C and Supplementary Figures 3C,D) as previously described in human mammary epithelial cells (Okoh et al., 2013). ROS-induced p85 phosphorylation would explain the ROS-induced increase in AKT phosphorylation (Figure 2C). Alternatively, ROS could inhibit phosphatases involved in PI3K/AKT regulation, for example PTEN, PP2A, or SHIP2. Such mechanism has been described in mouse skeletal muscle cells, embryonic fibroblasts, or HeLa cells (Lee et al., 2002; Zhang et al., 2007; Kim et al., 2018; Raman and Pervaiz, 2019).

### AKT Phosphorylation Is Upregulated by ROS-Mediated Downregulation of PTEN Activity

To elucidate the mechanism behind the downregulation of AKT phosphorylation by ROS, we focused on PTEN—a well-established PI3K antagonist (Leslie and Downes, 2002). Indeed, PTEN is probably involved in the attenuation of AKT phosphorylation in hPSCs because the downregulation of AKT phosphorylation can be reverted by PTEN silencing (Figure 6C and Supplementary Figures 6E,G). According to the literature, ROS-mediated cysteine oxidation renders PTEN inactive (Lee et al., 2002; Covey et al., 2007). Furthermore, we found the effect of PTEN silencing on AKT phosphorylation to be more profound in mild hypoxia than in 21% O_2_ (Figure 6C and Supplementary Figures 6E,G) and confirmed that ROS are capable of oxidizing PTEN in hPSCs (Figure 6E). ROS scavenging by GSH in 21% O_2_ also led to lower AKT phosphorylation, which was rescued by PTEN silencing (Figure 6D and Supplementary Figures 6F,G). These findings further support the hypothesis that ROS mediate downregulation of PTEN activity in hPSCs. AKT itself was also shown to be reversibly oxidized by ROS, which strengthens its PIP_3_ binding pocket, recruitment to the plasma membrane, and its activation (Su et al., 2019). Our results show that the possible AKT oxidation does not induce phosphorylation without the activity of PI3K (Figure 2D). Seemingly, the ROS-mediated strengthening of the PIP_3_ binding pocket on the AKT molecule (Su et al., 2019) cooperates with the reversible oxidation of PTEN to induce AKT phosphorylation.

PTEN was shown to be also regulated on the level of transcription, protein stability, and localization (Leslie and Downes, 2002). Since the total amount of PTEN remained stable when comparing 21 and 5% O_2_ (Figure 6A and Supplementary Figures 6A,B) and cells treated with GSH and H_2_O_2_ (Figure 6B and Supplementary Figures 6C,D), downregulation of AKT phosphorylation in hPSCs does not appear to be driven by changes in PTEN total amount or stability. The extent of PTEN phosphorylation can impact its localization (Vazquez et al., 2000), but similarly to the total amount, we did not detect any changes in PTEN phosphorylation in different oxygen tensions (Figure 6A and Supplementary Figures 6A,B) or in the presence of a different amount of ROS (Figure 6B and Supplementary Figures 6C,D). These data together suggest the involvement of only cysteine oxidation in the ROS-mediated regulation of PTEN activity in hPSCs.

We also analyzed the role of SH2-domain-containing inositol-5′-phosphatase (SHIP2). This enzyme is known to antagonize PI3K in a manner similar to PTEN and is described to be regulated by ROS (Zhang et al., 2007). As expected, 2-h inhibition of SHIP2 by AS19 had no effect on AKT phosphorylation in hPSCs (Figure 6F and Supplementary Figures 7A,B) since the effect of SHIP2 on AKT phosphorylation starts to appear after 6 h (Fafilek et al., 2018). While SHIP2 probably does play a role in long-term hypoxia-induced regulation of AKT, our data suggest that it is not involved in the immediate dynamic response to O_2_-induced ROS regulation of PI3K/AKT pathway in hPSCs.

ERK1/2 and AKT dephosphorylation have also been previously attributed to protein phosphatase 2A (PP2A). PP2A has been described to be negatively regulated by ROS, which might contribute to its activation in mild hypoxia (Rao and Clayton, 2002; Raman and Pervaiz, 2019). However, PP2A inhibition had no significant effect on AKT phosphorylation in hPSCs cultivated in 5 or 21% O_2_ (Figure 6G and Supplementary Figures 7C,D). Similarly, no changes in ERK1/2 phosphorylation were detected in our cells (Figure 1A and Supplementary Figures 1A,B). Therefore, PP2A does not seem to play a significant role in O_2_-induced ROS regulation of the PI3K/AKT pathway in hPSCs.

### HIF-1α Silencing Upregulates ROS/PI3K/AKT and Inhibits MAPK in Mild Hypoxia

HIFs 1 and 2 are master regulators of cellular adaptation to hypoxic conditions (Semenza, 2001; Keith et al., 2012). CoCl_2_ is a known hypoxia mimetic, stabilizing α subunits of HIFs, but it only transcriptionally activates HIF-1 (Befani et al., 2013). Mimicking hypoxia with CoCl_2_ led to stabilization of HIF-1α and downregulation of AKT phosphorylation (Figure 4A). Previously, HIF-1-deficient fibroblasts were shown to accumulate ROS (Zhang et al., 2008). Indeed, we found hESCs with downregulated HIF-1α to have significantly elevated ROS levels (Figure 4C) and upregulated AKT phosphorylation even in the absence of ligand FGF2 (Figure 4B and Supplementary Figure 4A). Together with our observations describing ROS-induced regulation of FGFR1 (Figure 3B) and PTEN (Figures 6C–E), these data suggest that HIF-1 driven downregulation of ROS regulates activation of FGFR and PTEN inactivation in mild hypoxia. This is strikingly different from the mechanism in mESCs, where the ROS-mediated downregulation of PI3K/AKT and MAPK pathways is HIF-1 independent (Kučera et al., 2017). It is also important to mention that ROS probably influence other pathways converging on PI3K/AKT through different RTKs (Chiarugi and Buricchi, 2007; Kruk et al., 2013) and different phosphatases (Chiarugi and Cirri, 2003; Esposito et al., 2003; Chiarugi and Buricchi, 2007; Raman and Pervaiz, 2019), for example, the insulin or insulin-like signaling pathway (Papaconstantinou, 2009; Dalton, 2013).

### MAPK Downregulate PI3K/AKT and FGFR1 Phosphorylation via ROS

The silencing of HIF-1α expression led to downregulation of ERK1/2 phosphorylation and simultaneous upregulation of AKT phosphorylation in hPSCs (Figure 4B and Supplementary Figure 4A). MAPK was previously shown to downregulate PI3K/AKT regulating the adaptor protein Gab1 (Yu et al., 2002) or by recruitment of PTEN to the plasma membrane (Zmajkovicova et al., 2013). In hPSCs, MEK1/2 inhibition also upregulated AKT phosphorylation (Figure 5A and Supplementary Figures 5A,B). MEK1/2 inhibition in mild hypoxia was also accompanied by an elevation of ROS (Figure 5B). Even though MEK1/2 inhibition-mediated elevation of ROS was not statistically significant in 21% O_2_, AKT phosphorylation was rescued by downregulation of ROS (Figure 5C and Supplementary Figures 5C,D). These data implicate that MAPK attenuates PI3K/AKT via ROS as second messengers. Moreover, MEK1/2 inhibition led to upregulation of FGFR1 phosphorylation in 21% O_2_, which can be prevented by ROS scavenging (Figure 5D, Supplementary Figures 5C,E). Even though our results stem from an artificial MEK1/2–ERK1/2 manipulation, MEK1/2–ERK1/2-induced inhibition of ROS suggest this mechanism may serve as a negative feedback loop from MAPK toward FGFR1. A similar negative feedback loop involving p38 has already been described in the literature (Zakrzewska et al., 2019). The detailed mechanism by which MEK1/2–ERK1/2 contributes to the ROS downregulation is so far unclear, but the MAPK pathway was shown to play an important role in metabolic reprogramming and upregulation of glycolysis (Papa et al., 2019). Glycolysis provides its intermediates to the pentose phosphate pathway (PPP), a significant NADPH source. NADPH is known to contribute to the ROS scavenging by providing its reductive potential to GSH and thioredoxins, consequently utilized to neutralize ROS (Hanschmann et al., 2013). It is, therefore, possible that MEK1/2–ERK1/2 may downregulate ROS in hPSCs via upregulation of NADPH production in PPP.

### ROS Scavenging Upregulates Pluripotency Markers

Several studies have shown that a hypoxic environment improves hPSCs pluripotency (Ezashi et al., 2005; Mathieu et al., 2013), which was linked to HIFs-induced pluripotency gene expression (Forristal et al., 2010; Mathieu et al., 2014). Mild hypoxia-induced ROS downregulation might contribute to the pluripotency maintenance, at least according to our data showing that ROS scavenging with GSH in 21% O_2_ upregulates pluripotency markers on levels similar to those observed in 5% O_2_ (Figures 7A,B). This is in concert with a previous study showing that ROS are able to induce hPSCs differentiation (Ji et al., 2010). The role of PI3K/AKT attenuation due to ROS downregulation in pluripotency maintenance is unclear, but it could be an interesting topic of further studies, since PI3K/AKT signaling directs cell fate decision upon differentiation priming (Yu and Cui, 2016).

Another possible mechanism for ROS-mediated regulation of pluripotency is integrative nuclear FGFR1 signaling (INFS), which was shown to downregulate the expression of core pluripotency genes and to be instrumental in neural differentiation (Terranova et al., 2015). INFS-induced localization of FGFR1 in the nucleus is induced by activation of cell surface receptors (Stachowiak et al., 2007) and may include interaction with p85α (Dunham et al., 2004). Cell surface FGFR1 phosphorylation can be promoted by ROS as shown by us (Figures 3A,B) and others (Chiarugi and Cirri, 2003; Chiarugi and Buricchi, 2007). Similarly, the nuclear localization of FGFR2 negatively regulates HIFs in prostate cancer (Lee et al., 2019). We observed both the cytoplasmic and nuclear localization of FGFR1 in hPSCs using ICC (Supplementary Figure 8). We did not observe downregulation of FGFR1 nuclear localization after GSH or 5% O_2_ treatment, but it is possible that this mechanism is employed in hPSCs but is under the ICC detection threshold. Such a mechanism would lead to FGFR1-activation-dependent downregulation of the pluripotency signaling network in response to ROS and consequent differentiation.

**Figure 8:**
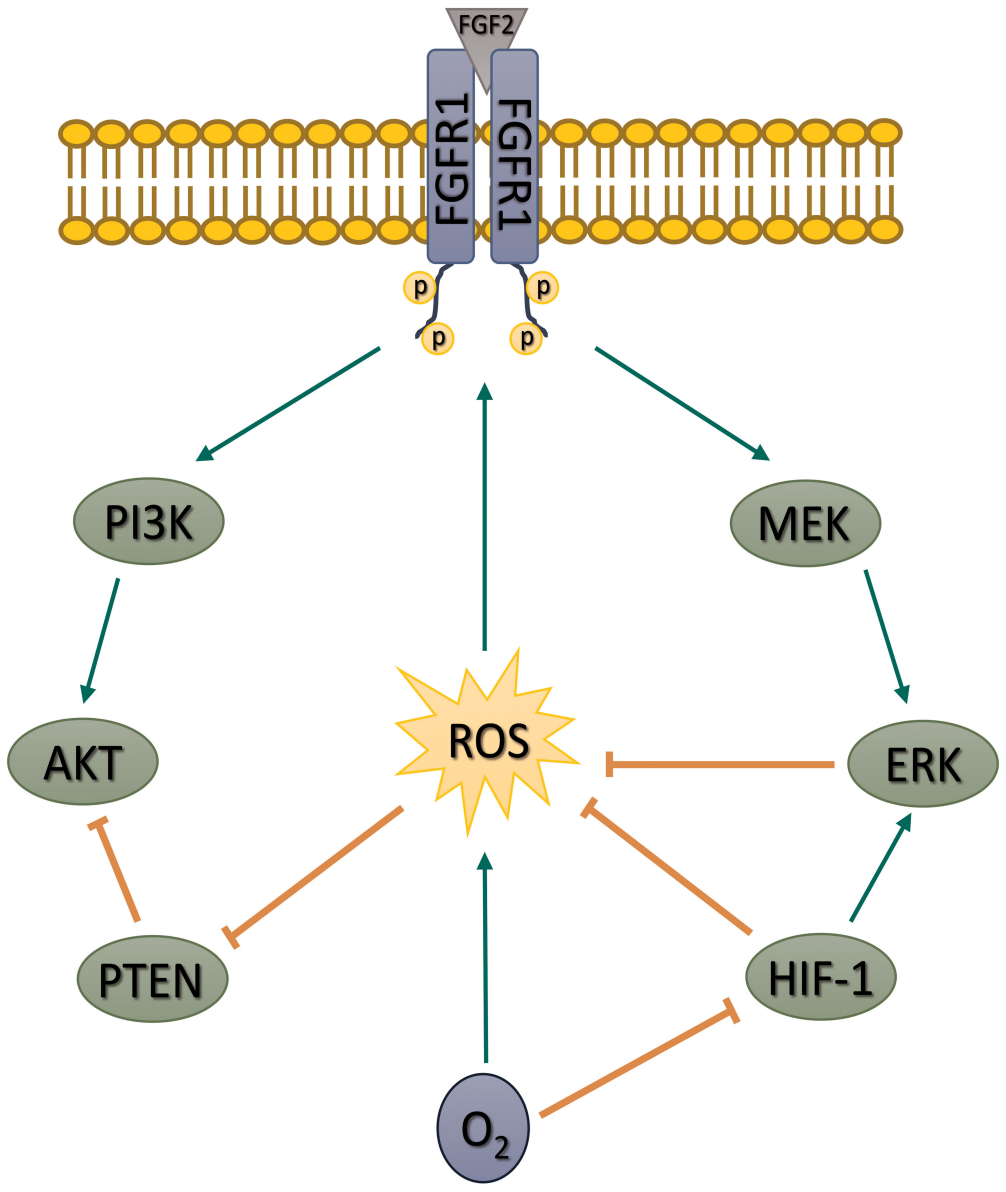
Schematic depiction of our proposed model of the role of ROS in the regulation of FGF2-induced AKT phosphorylation in hPSCs. Based on our artificial manipulations of the MEK1/2–ERK1/2 pathway and ROS levels, we propose that MEK1/2–ERK1/2 pathway and mild hypoxia (5% O_2_) downregulate AKT phosphorylation using ROS as second messengers. We further show that ROS contribute to AKT phosphorylation by FGFR1 transactivation and inhibition of PTEN activity in hPSCs. ROS, reactive oxygen species; FGF2, fibroblast growth factor 2; AKT, protein kinase B; hPSCs, human pluripotent stem cells; MAPK, mitogen-activated protein kinase; FGFR1, fibroblast growth factor receptor 1; PTEN, phosphatase and tensin homolog deleted on chromosome 10; MEK1/2, mitogen-activated protein kinase kinase 1 and 2; ERK1/2, extracellular signal-regulated kinase 1 and 2; HIF-1, hypoxia-inducible factor 1; PI3K, phosphatydylinositol-4,5-bisphosphate 3-kinase.

Taken together, our data show that the PI3K/AKT pathway in hPSCs is upregulated by ROS. The upregulation is secured by an increase in FGFR1-activating Tyr653/654 phosphorylation, PI3K p85 phosphorylation, and a decrease in PTEN activity. We further show that MAPK pathway and also mild hypoxia (in HIF-1-dependent manner) attenuate ROS and thus downregulate but do not completely shut off the PI3K/AKT signaling (Figure 8)—a pathway essential for hPSCs pluripotency maintenance, self-renewal, and cell fate decision. Such mechanism differs from observations made in mESCs (Kučera et al., 2017), possibly due to a different pluripotency status. Pluripotency maintenance and iPSCs reprogramming was shown to benefit from mild hypoxia, an environment native to the blastocyst; therefore, it is counterintuitive that it leads to PI3K/AKT downregulation. Higher PI3K/AKT activity in 21% O_2_ could theoretically compensate for the missing hypoxia-induced pluripotency signaling. On the other hand, the PI3K/AKT pathway has also been implicated in the regulation of differentiation, as summarized by Yu and Cui (Yu and Cui, 2016). It seems that a precise balance in signaling pathways activity helps to maintain pluripotency, and swings or disbalances in their activity induced by signaling molecules and external factors can affect the fragile balance between pluripotency and differentiation. We hypothesize that a mildly hypoxic environment via HIF-1 and also the MEK1/2–ERK1/2 pathway might help to maintain such balance in PI3K/AKT (and possibly other ROS-sensitive pathways) activity in hPSCs by controlling levels of second messengers—ROS. We propose that introducing ROS control into the hPSCs maintenance and differentiation protocols might thus lead to their significant improvement (Figures 7A,B). Because of the similarity between hPSCs and cancer stem cells and because the PI3K/AKT pathway often undergoes an oncogenic transformation, our results might also help deepen the current understanding of cancer biology.

## Data Availability Statement

The original contributions presented in the study are included in the article/Supplementary Materials, further inquiries can be directed to the corresponding author/s.

## Author Contributions

PF performed cell cultivation, WB with analysis, ROS analysis, and ICC analysis. DB performed qRT-PCR and analyzed the data. KH performed cell cultivation and Western blot analysis. MŠ performed WB and ICC. VR and PF wrote the manuscript and designed all experiments. All authors contributed to the article and approved the submitted version.

## Conflict of Interest

The authors declare that the research was conducted in the absence of any commercial or financial relationships that could be construed as a potential conflict of interest.

## Abbreviations

AKT: protein kinase B
AS19: AS1938909
CM–: conditioned human embryonic stem cell media without FGF2 supplementation
CM+: conditioned human embryonic stem cell media with FGF2 supplementation
Ctrl: control
ERK1/2: extracellular signal-regulated kinase 1 and 2
ESCs: embryonic stem cells
esiRNA: endoribonuclease-prepared short-interfering RNA
FGF2: fibroblast growth factor 2
FGFR1: fibroblast growth factor receptor 1
GAPDH: glyceraldehyde 3-phosphate dehydrogenase
GSH: glutathione
hES media: human embryonic stem cell media
hESCs: human embryonic stem cells
HIF-1: hypoxia-inducible factor 1
HIFs: hypoxia-inducible factors
hPSCs: human pluripotent stem cells
INFS: integrative nuclear FGFR1 signaling
iPSCs: induced pluripotent stem cells
MAPK: mitogen mitogen-activated protein kinase
Matrigel: Matrigel hESC-qualified Matrix
MEK1/2: mitogen mitogen-activated protein kinase kinase 1 and 2
mESCs: mouse embryonic stem cells
OKA: okadaic acid
pAKT: phosphorylated protein kinase B
PBS: phosphate buffered saline
PCNA: proliferating cell nuclear antigen
PD03: PD0325901
PD18: PD184161
PIP_3_: phosphatidylinositol 3,4,5-triphosphate
PPP: pentose phosphate pathway
pERK: phosphorylated extracellular signal-regulated kinase 1 and 2
pFGFR1: phosphorylated fibroblast growth factor 1
PI3K: phosphatydylinositol-4,5-bisphosphate 3-kinase
PI3K p85: p85 subunit of phosphatydylinositol-4,5-bisphosphate 3-kinase
PP1: protein phosphatase 1
PP2A: protein phosphatase 2A
pPI3K p85: phosphorylated p85 subunit of phosphatydylinositol-4,5-bisphosphate 3-kinase
pPTEN: phosphorylated phosphatase and tensin homolog deleted on chromosome 10
PTEN: phosphatase and tensin homolog deleted on chromosome 10
ROS: reactive oxygen species
RTKs: receptor tyrosine kinases
Scr: non-targeting short-interfering RNA
SHIP2: SH2 domain-containing inositol-5′-phosphatase 2
TBS-T: tris buffered saline with Tween 20
qRT-PCR: quantitative real-time PCR
Vin: vinculin
WB: Western blot
αTu: alpha tubulin.

## References

[B1] Adewumi O. Aflatoonian B. Ahrlund-Richter L. Amit M. Andrews P. W. Beighton G.. (2007). Characterization of human embryonic stem cell lines by the international stem cell initiative. Nat. Biotechnol. 25, 803–816. 10.1038/nbt1318 17572666

[B2] Aksamitiene E. Kiyatkin A. Kholodenko B. N. (2012). Cross-talk between mitogenic Ras/MAPK and survival PI3K/Akt pathways: a fine balance. Biochem. Soc. Trans. 40, 139–146. 10.1042/BST20110609 22260680

[B3] Alvarez-Tejado M. Naranjo-Suárez S. Jiménez C. Carrera A. C. Landázuri M. O. Del Peso L. (2001). Hypoxia induces the activation of the phosphatidylinositol 3-kinase/Akt cell survival pathway in PC12 cells. Protective role in apoptosis. J. Biol. Chem. 276, 22368–22374. 10.1074/jbc.M011688200 11294857

[B4] Armstrong L. Hughes O. Yung S. Hyslop L. Stewart R. Wappler I.. (2006). The role of PI3K/AKT, MAPK/ERK and NFκβ signalling in the maintenance of human embryonic stem cell pluripotency and viability highlighted by transcriptional profiling and functional analysis. Hum. Mol. Genet. 15, 1894–1913. 10.1093/hmg/ddl112 16644866

[B5] Befani C. Mylonis I. Gkotinakou I. M. Georgoulias P. Hu C. J. Simos G.. (2013). Cobalt stimulates HIF-1-dependent but inhibits HIF-2-dependent gene expression in liver cancer cells. Int. J. Biochem. Cell Biol. 45, 2359–2368. 10.1016/j.biocel.2013.07.025 23958427 PMC3855297

[B6] Chiarugi P. Buricchi F. (2007). Protein tyrosine phosphorylation and reversible oxidation: two cross-talking posttranslation modifications. Antioxid. Redox Signal. 9, 1–24. 10.1089/ars.2007.9.1 17115885

[B7] Chiarugi P. Cirri P. (2003). Redox regulation of protein tyrosine phosphatases during receptor tyrosine kinase signal transduction. Trends Biochem. Sci. 28, 509–514. 10.1016/S0968-0004(03)00174-9 13678963

[B8] Covello K. L. Kehler J. Yu H. Gordan J. D. Arsham A. M. Hu C. J.. (2006). HIF-2α regulates Oct-4: effects of hypoxia on stem cell function, embryonic development, and tumor growth. Genes Dev. 20, 557–570. 10.1101/gad.1399906 16510872 PMC1410808

[B9] Covey T. M. Edes K. Fitzpatrick F. A. (2007). Akt activation by arachidonic acid metabolism occurs via oxidation and inactivation of PTEN tumor suppressor. Oncogene 26, 5784–5792. 10.1038/sj.onc.1210391 17369849

[B10] Dalton S. (2013). Signaling networks in human pluripotent stem cells. Curr. Opin. Cell Biol. 25, 241–246. 10.1016/j.ceb.2012.09.005 23092754 PMC3570582

[B11] Dunham S. M. Pudavar H. E. Prasad P. N. Stachowiak M. K. (2004). Cellular signaling and protein-protein interactions studied using fluorescence recovery after photobleaching. J. Phys. Chem. B 108, 10540–10546. 10.1021/jp0400972

[B12] Dvorak P. Dvorakova D. Koskova S. Vodinska M. Najvirtova M. Krekac D.. (2005). Expression and potential role of fibroblast growth factor 2 and its receptors in human embryonic stem cells. Stem Cells 23, 1200–1211. 10.1634/stemcells.2004-0303 15955829

[B13] Eiselleova L. Matulka K. Kriz V. Kunova M. Schmidtova Z. Neradil J.. (2009). A complex role for FGF-2 in self-renewal, survival, and adhesion of human embryonic stem cells. Stem Cells 27, 1847–1857. 10.1002/stem.128 19544431 PMC2798073

[B14] Esposito F. Chirico G. Gesualdi N. M. Posadas I. Ammendola R. Russo T.. (2003). Protein kinase B activation by reactive oxygen species is independent of tyrosine kinase receptor phosphorylation and requires Src activity. J. Biol. Chem. 278, 20828–20834. 10.1074/jbc.M211841200 12682076

[B15] Ezashi T. Das P. Roberts R. M. (2005). Low O_2_ tensions and the prevention of differentiation of hES cells. Proc. Natl. Acad. Sci. U.S.A. 102, 4783–4788. 10.1073/pnas.0501283102 15772165 PMC554750

[B16] Fafilek B. Balek L. Bosakova M. K. Varecha M. Nita A. Gregor T.. (2018). The inositol phosphatase SHIP2 enables sustained ERK activation downstream of FGF receptors by recruiting Src kinases. Sci. Signal. 11:eaap8608. 10.1126/scisignal.aap8608 30228226 PMC12677889

[B17] Fischer B. Bavister B. D. (1993). Oxygen tension in the oviduct and uterus of rhesus monkeys, hamsters and rabbits. J. Reprod. Fertil. 99, 673–679. 10.1530/jrf.0.0990673 8107053

[B18] Forristal C. E. Wright K. L. Hanley N. A. Oreffo R. O. C. Houghton F. D. (2010). Hypoxia inducible factors regulate pluripotency and proliferation in human embryonic stem cells cultured at reduced oxygen tensions. Reproduction 139, 85–97. 10.1530/REP-09-0300 19755485 PMC2791494

[B19] Genestra M. (2007). Oxyl radicals, redox-sensitive signalling cascades and antioxidants. Cell. Signal. 19, 1807–1819. 10.1016/j.cellsig.2007.04.009 17570640

[B20] Haghighi F. Dahlmann J. Nakhaei-Rad S. Lang A. Kutschka I. Zenker M.. (2018). BFGF-mediated pluripotency maintenance in human induced pluripotent stem cells is associated with NRAS-MAPK signaling 06 biological sciences 0601 biochemistry and cell biology. Cell Commun. Signal. 16:96. 10.1186/s12964-018-0307-1 30518391 PMC6282345

[B21] Hanschmann E. M. Godoy J. R. Berndt C. Hudemann C. Lillig C. H. (2013). Thioredoxins, glutaredoxins, and peroxiredoxins-molecular mechanisms and health significance: from cofactors to antioxidants to redox signaling. Antioxid. Redox Signal. 19, 1539–1605. 10.1089/ars.2012.4599 23397885 PMC3797455

[B22] Hossini A. M. Quast A. S. Plötz M. Grauel K. Exner T. Küchler J.. (2016). PI3K/AKT signaling pathway is essential for survival of induced pluripotent stem cells. PLoS ONE 11:e0154770. 10.1371/journal.pone.0154770 27138223 PMC4854383

[B23] Hung S.-C. Pochampally R. R. Chen S.-C. Hsu S.-C. Prockop D. J. (2007). Angiogenic effects of human multipotent stromal cell conditioned medium activate the PI3K-akt pathway in hypoxic endothelial cells to inhibit apoptosis, increase survival, and stimulate angiogenesis. Stem Cells 25, 2363–2370. 10.1634/stemcells.2006-0686 17540857

[B24] Jelinkova S. Fojtik P. Kohutova A. Vilotic A. Marková L. Pesl M.. (2019). Dystrophin deficiency leads to genomic instability in human pluripotent stem cells via NO synthase-induced oxidative stress. Cells 8:53. 10.3390/cells8010053 30650618 PMC6356905

[B25] Ji A. R. Ku S. Y. Cho M. S. Kim Y. Y. Kim Y. J. Oh S. K.. (2010). Reactive oxygen species enhance differentiation of human embryonic stem cells into mesendodermal lineage. Exp. Mol. Med. 42, 175–186. 10.3858/emm.2010.42.3.018 20164681 PMC2845002

[B26] Keith B. Johnson R. S. Simon M. C. (2012). HIF1 α and HIF2 α: sibling rivalry in hypoxic tumour growth and progression. Nat. Rev. Cancer 12, 9–22. 10.1038/nrc3183 22169972 PMC3401912

[B27] Khacho M. Clark A. Svoboda D. S. Azzi J. MacLaurin J. G. Meghaizel C.. (2016). Mitochondrial dynamics impacts stem cell identity and fate decisions by regulating a nuclear transcriptional program. Cell Stem Cell 19, 232–247. 10.1016/j.stem.2016.04.015 27237737

[B28] Kim J. H. Choi T. G. Park S. Yun H. R. Nguyen N. N. Y. Jo Y. H.. (2018). Mitochondrial ROS-derived PTEN oxidation activates PI3K pathway for mTOR-induced myogenic autophagy. Cell Death Differ. 25, 1921–1937. 10.1038/s41418-018-0165-9 30042494 PMC6219511

[B29] Kruk J. S. Vasefi M. S. Heikkila J. J. Beazely M. A. (2013). Reactive oxygen species are required for 5-HT-induced transactivation of neuronal platelet-derived growth factor and TrkB receptors, but not for ERK1/2 activation. PLoS ONE 8:e77027. 10.1371/journal.pone.0077027 24086766 PMC3785432

[B30] Krutá M. Šeneklová M. Raška J. Salykin A. Zerzánková L. Pešl M.. (2014). Mutation frequency dynamics in hprt locus in culture-adapted human embryonic stem cells and induced pluripotent stem cells correspond to their differentiated counterparts. Stem Cells Dev. 23, 2443–2454. 10.1089/scd.2013.0611 24836366 PMC4186764

[B31] Kučera J. Netušilová J. Sladeček S. Lánová M. Vašíček O. Štefková K.. (2017). Hypoxia downregulates MAPK/ERK but not STAT3 signaling in ROS-dependent and HIF-1-independent manners in mouse embryonic stem cells. Oxid. Med. Cell. Longev. 2017:438647. 10.1155/2017/4386947 28819544 PMC5551543

[B32] Kunath T. Saba-El-Leil M. K. Almousailleakh M. Wray J. Meloche S. Smith A. (2007). FGF stimulation of the Erk1/2 signalling cascade triggers transition of pluripotent embryonic stem cells from self-renewal to lineage commitment. Development 134, 2895–2902. 10.1242/dev.02880 17660198

[B33] Lee H. H. Chang C. C. Shieh M. J. Wang J. P. Chen Y. Te Y.. (2013). Hypoxia enhances chondrogenesis and prevents terminal differentiation through pi3k/akt/foxo dependent anti-apoptotic effect. Sci. Rep. 3:2683. 10.1038/srep02683 24042188 PMC3775095

[B34] Lee J. E. Shin S. H. Shin H. W. Chun Y. S. Park J. W. (2019). Nuclear FGFR2 negatively regulates hypoxia-induced cell invasion in prostate cancer by interacting with HIF-1 and HIF-2. Sci. Rep. 9:3480. 10.1038/s41598-019-39843-6 30837551 PMC6401139

[B35] Lee S. R. Yang K. S. Kwon J. Lee C. Jeong W. Rhee S. G. (2002). Reversible inactivation of the tumor suppressor PTEN by H_2_O_2_. J. Biol. Chem. 277, 20336–20342. 10.1074/jbc.M111899200 11916965

[B36] Leslie N. R. Downes C. P. (2002). PTEN: the down side of PI 3-kinase signalling. Cell. Signal. 14, 285–295. 10.1016/S0898-6568(01)00234-0 11858936

[B37] Levenstein M. E. Ludwig T. E. Xu R.-H. Llanas R. A. VanDenHeuvel-Kramer K. Manning D.. (2006). Basic fibroblast growth factor support of human embryonic stem cell self-renewal. Stem Cells 24, 568–574. 10.1634/stemcells.2005-0247 16282444 PMC4615709

[B38] Li J. Wang G. Wang C. Zhao Y. Zhang H. Tan Z.. (2007). MEK/ERK signaling contributes to the maintenance of human embryonic stem cell self-renewal. Differentiation 75, 299–307. 10.1111/j.1432-0436.2006.00143.x 17286604

[B39] Maddalena L. A. Selim S. M. Fonseca J. Messner H. McGowan S. Stuart J. A. (2017). Hydrogen peroxide production is affected by oxygen levels in mammalian cell culture. Biochem. Biophys. Res. Commun. 493, 246–251. 10.1016/j.bbrc.2017.09.037 28899780

[B40] Mathieu J. Zhang Z. Nelson A. Lamba D. A. Reh T. A. Ware C.. (2013). Hypoxia induces re-entry of committed cells into pluripotency. Stem Cells 31, 1737–1748. 10.1002/stem.1446 23765801 PMC3921075

[B41] Mathieu J. Zhou W. Xing Y. Sperber H. Ferreccio A. Agoston Z.. (2014). Hypoxia-inducible factors have distinct and stage-specific roles during reprogramming of human cells to pluripotency. Cell Stem Cell 14, 592–605. 10.1016/j.stem.2014.02.012 24656769 PMC4028142

[B42] Mendoza M. C. Er E. E. Blenis J. (2011). The Ras-ERK and PI3K-mTOR pathways: cross-talk and compensation. Trends Biochem. Sci. 36, 320–328. 10.1016/j.tibs.2011.03.006 21531565 PMC3112285

[B43] Miyamoto L. Yagi Y. Hatano A. Kawazoe K. Ishizawa K. Minakuchi K.. (2015). Spontaneously hyperactive MEK-Erk pathway mediates paradoxical facilitation of cell proliferation in mild hypoxia. Biochim. Biophys. Acta Gen. Subj. 1850, 640–646. 10.1016/j.bbagen.2014.12.006 25497211

[B44] Okazaki K. Maltepe E. (2006). Oxygen, epigenetics and stem cell fate. Regen. Med. 1, 71–83. 10.2217/17460751.1.1.71 17465821

[B45] Okoh V. O. Felty Q. Parkash J. Poppiti R. Roy D. (2013). Reactive oxygen species via redox signaling to PI3K/AKT pathway contribute to the malignant growth of 4-hydroxy estradiol-transformed mammary epithelial cells. PLoS ONE 8:e54206. 10.1371/journal.pone.0054206 23437041 PMC3578838

[B46] Östman A. Frijhoff J. Sandin Å. Böhmer F. D. (2011). Regulation of protein tyrosine phosphatases by reversible oxidation. J. Biochem. 150, 345–356. 10.1093/jb/mvr104 21856739

[B47] Papa S. Choy P. M. Bubici C. (2019). The ERK and JNK pathways in the regulation of metabolic reprogramming. Oncogene 38, 2223–2240. 10.1038/s41388-018-0582-8 30487597 PMC6398583

[B48] Papaconstantinou J. (2009). Insulin/IGF-1 and ROS signaling pathway cross-talk in aging and longevity determination. Mol. Cell. Endocrinol. 299, 89–100. 10.1016/j.mce.2008.11.025 19103250 PMC2873688

[B49] Raman D. Pervaiz S. (2019). Redox inhibition of protein phosphatase PP2A: potential implications in oncogenesis and its progression. Redox Biol. 27:101105. 10.1016/j.redox.2019.101105 30686777 PMC6859563

[B50] Rao R. K. Clayton L. W. (2002). Regulation of protein phosphatase 2A by hydrogen peroxide and glutathionylation. Biochem. Biophys. Res. Commun. 293, 610–616. 10.1016/S0006-291X(02)00268-1 12054646

[B51] Rhee S. G. (2006). H_2_O_2_, a necessary evil for cell signaling. Science 312, 1882–1883. 10.1126/science.1130481 16809515

[B52] Semenza G. L. (2001). HIF-1 and mechanisms of hypoxia sensing. Curr. Opin. Cell Biol. 13, 167–71. 10.1016/S0955-0674(00)00194-0 11248550

[B53] Shiojima I. Walsh K. (2002). Role of Akt signaling in vascular homeostasis and angiogenesis. Circ. Res. 90, 1243–1250. 10.1161/01.RES.0000022200.71892.9F 12089061

[B54] Singh A. M. Reynolds D. Cliff T. Ohtsuka S. Mattheyses A. L. Sun Y.. (2012). Signaling network crosstalk in human pluripotent cells: a Smad2/3-regulated switch that controls the balance between self-renewal and differentiation. Cell Stem Cell 10, 312–326. 10.1016/j.stem.2012.01.014 22385658 PMC3294294

[B55] Stachowiak M. K. Maher P. A. Stachowiak E. K. (2007). Integrative nuclear signaling in cell development - A role for FGF receptor-1. DNA Cell Biol. 26, 811–826. 10.1089/dna.2007.0664 18021009

[B56] Su Z. Burchfield J. G. Yang P. Humphrey S. J. Yang G. Francis D.. (2019). Global redox proteome and phosphoproteome analysis reveals redox switch in Akt. Nat. Commun. 10:5486. 10.1038/s41467-019-13114-4 31792197 PMC6889415

[B57] Takahashi K. Tanabe K. Ohnuki M. Narita M. Ichisaka T. Tomoda K.. (2007). Induction of pluripotent stem cells from adult human fibroblasts by defined factors. Cell 131, 861–872. 10.1016/j.cell.2007.11.019 18035408

[B58] Terranova C. Narla S. T. Lee Y. W. Bard J. Parikh A. Stachowiak E. K.. (2015). Global developmental gene programing involves a nuclear form of fibroblast growth factor receptor-1 (FGFR1). PLoS ONE 10:e0123380. 10.1371/journal.pone.0123380 25923916 PMC4414453

[B59] Vazquez F. Grossman S. R. Takahashi Y. Rokas M. V. Nakamura N. Sellers W. R. (2001). Phosphorylation of the PTEN tail acts as an inhibitory switch by preventing its recruitment into a protein complex. J. Biol. Chem. 276, 48627–48630. 10.1074/jbc.C100556200 11707428

[B60] Vazquez F. Ramaswamy S. Nakamura N. Sellers W. R. (2000). Phosphorylation of the PTEN tail regulates protein stability and function. Mol. Cell. Biol. 20, 5010–5018. 10.1128/MCB.20.14.5010-5018.2000 10866658 PMC85951

[B61] Wang X. Q. Lo C. M. Chen L. Ngan E. S. W. Xu A. Poon R. Y. C. (2017). CDK1-PDK1-PI3K/Akt signaling pathway regulates embryonic and induced pluripotency. Cell Death Differ. 24, 38–48. 10.1038/cdd.2016.84 27636107 PMC5260505

[B62] Ward P. S. Thompson C. B. (2012). Metabolic reprogramming: a cancer hallmark even warburg did not anticipate. Cancer Cell 21, 297–308. 10.1016/j.ccr.2012.02.014 22439925 PMC3311998

[B63] Xu C. Rosler E. Jiang J. Lebkowski J. S. Gold J. D. O'Sullivan C.. (2005a). Basic fibroblast growth factor supports undifferentiated human embryonic stem cell growth without conditioned medium. Stem Cells 23, 315–323. 10.1634/stemcells.2004-0211 15749926

[B64] Xu R. H. Peck R. M. Li D. S. Feng X. Ludwig T. Thomson J. A. (2005b). Basic FGF and suppression of BMP signaling sustain undifferentiated proliferation of human ES cells. Nat. Methods 2, 185–190. 10.1038/nmeth744 15782187

[B65] Yoshida Y. Takahashi K. Okita K. Ichisaka T. Yamanaka S. (2009). Hypoxia enhances the generation of induced pluripotent stem cells. Cell Stem Cell 5, 237–241. 10.1016/j.stem.2009.08.001 19716359

[B66] Yu C. F. Liu Z. X. Cantley L. G. (2002). ERK negatively regulates the epidermal growth factor-mediated interaction of Gab1 and the phosphatidylinositol 3-kinase. J. Biol. Chem. 277, 19382–19388. 10.1074/jbc.M200732200 11896055

[B67] Yu J. S. L. Cui W. (2016). Proliferation, survival and metabolism: the role of PI3K/AKT/ mTOR signalling in pluripotency and cell fate determination. Development 143, 3050–3060. 10.1242/dev.137075 27578176

[B68] Yu P. Pan G. Yu J. Thomson J. A. (2011). FGF2 sustains NANOG and switches the outcome of BMP4-induced human embryonic stem cell differentiation. Cell Stem Cell 8, 326–334. 10.1016/j.stem.2011.01.001 21362572 PMC3052735

[B69] Zakrzewska M. Opalinski L. Haugsten E. M. Otlewski J. Wiedlocha A. (2019). Crosstalk between p38 and Erk 1/2 in downregulation of FGF1-induced signaling. Int. J. Mol. Sci. 20:1826. 10.3390/ijms20081826 31013829 PMC6514807

[B70] Zeng L. Zhou H. Y. Tang N. N. Zhang W. F. He G. J. Hao B.. (2016). Wortmannin influences hypoxia-inducible factor-1 alpha expression and glycolysis in esophageal carcinoma cells. World J. Gastroenterol. 22, 4868–4880. 10.3748/wjg.v22.i20.4868 27239113 PMC4873879

[B71] Zhang H. Bosch-Marce M. Shimoda L. A. Yee S. T. Jin H. B. Wesley J. B.. (2008). Mitochondrial autophagy is an HIF-1-dependent adaptive metabolic response to hypoxia. J. Biol. Chem. 283, 10892–10903. 10.1074/jbc.M800102200 18281291 PMC2447655

[B72] Zhang J. Liu Z. Rasschaert J. Blero D. Deneubourg L. Schurmans S.. (2007). SHIP2 controls PtdIns(3,4,5)P3 levels and PKB activity in response to oxidative stress. Cell. Signal. 19, 2194–2200. 10.1016/j.cellsig.2007.06.022 17643961

[B73] Zhang J. Wang X. Vikash V. Ye Q. Wu D. Liu Y.. (2016). ROS and ROS-mediated cellular signaling. Oxid. Med. Cell. Longev. 2016:4350965. 10.1155/2016/4350965 26998193 PMC4779832

[B74] Zhang L. Liu Q. Lu L. Zhao X. Gao X. Wang Y. (2011). Astragaloside IV stimulates angiogenesis and increases hypoxia-inducible factor-1α accumulation via phosphatidylinositol 3-kinase/akt pathway. J. Pharmacol. Exp. Ther. 338, 485–491. 10.1124/jpet.111.180992 21576377

[B75] Zhang Y. Yang J. H. (2013). Activation of the PI3K/Akt pathway by oxidative stress mediates high glucose-induced increase of adipogenic differentiation in primary rat osteoblasts. J. Cell. Biochem. 114, 2595–2602. 10.1002/jcb.24607 23757055

[B76] Zmajkovicova K. Jesenberger V. Catalanotti F. Baumgartner C. Reyes G. Baccarini M. (2013). MEK1 is required for PTEN membrane recruitment, AKT regulation, and the maintenance of peripheral tolerance. Mol. Cell 50, 43–55. 10.1016/j.molcel.2013.01.037 23453810 PMC3625979

