# Supplementary material for: Both Hypoxia-Inducible Factor 1 and MAPK Signaling Pathway Attenuate PI3K/AKT via Suppression of Reactive Oxygen Species in Human Pluripotent Stem Cells
Source: Front Cell Dev Biol. 2021 Jan 21;8:607444. doi: 10.3389/fcell.2020.607444 (PMC7859355; doi:10.3389/fcell.2020.607444)

Figure 1A

Membranes were developed using Immobilon Western Chemiluminiscent HRP Substrate and SYNGENE G:Box Chemi

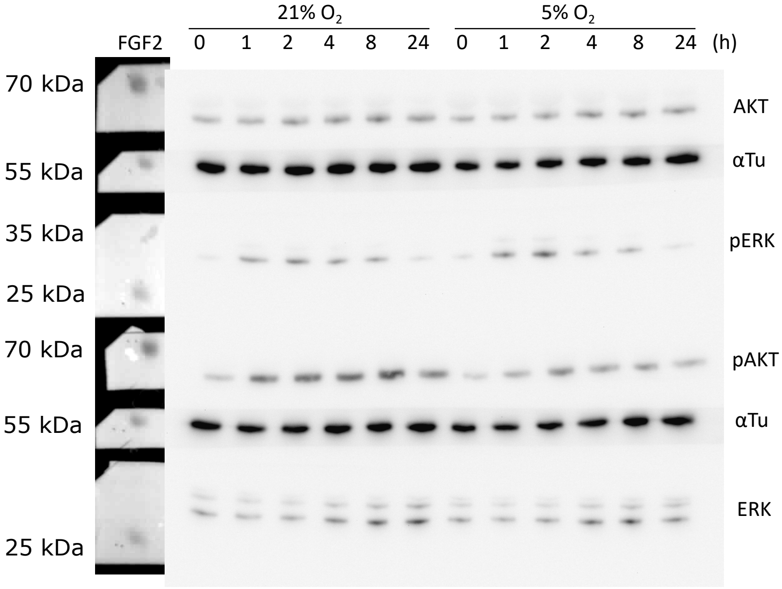

Figure 2C

Membranes were developed using Immobilon Western Chemiluminiscent HRP Substrate and SYNGENE G:Box Chemi

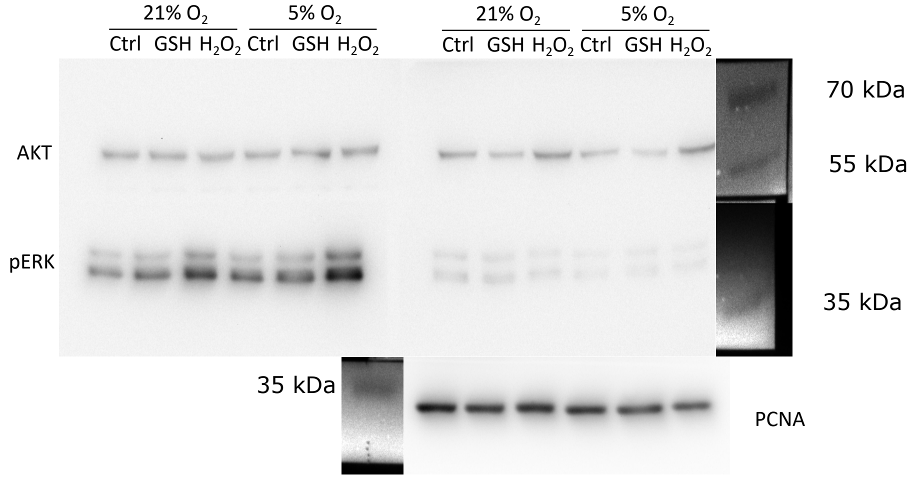

Figure 3A

Membranes were developed using Immobilon Western Chemiluminiscent HRP Substrate and SYNGENE G:Box Chemi

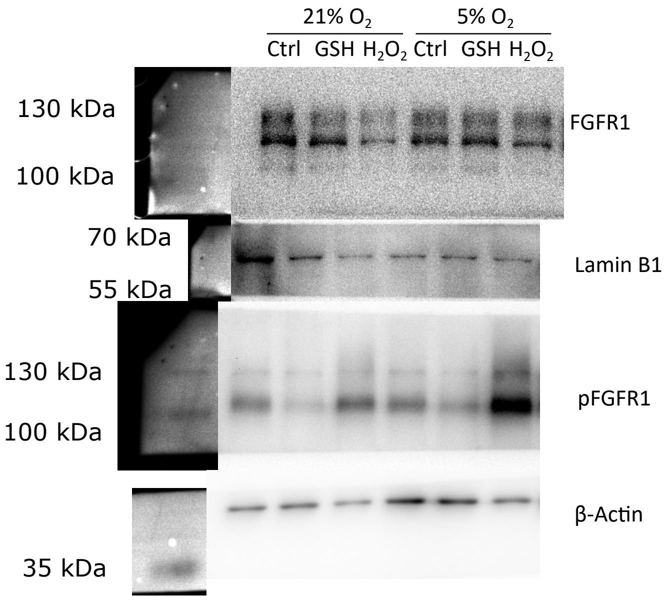

Figure 3C

Membranes were developed using Immobilon Western Chemiluminiscent HRP Substrate and SYNGENE G:Box Chemi

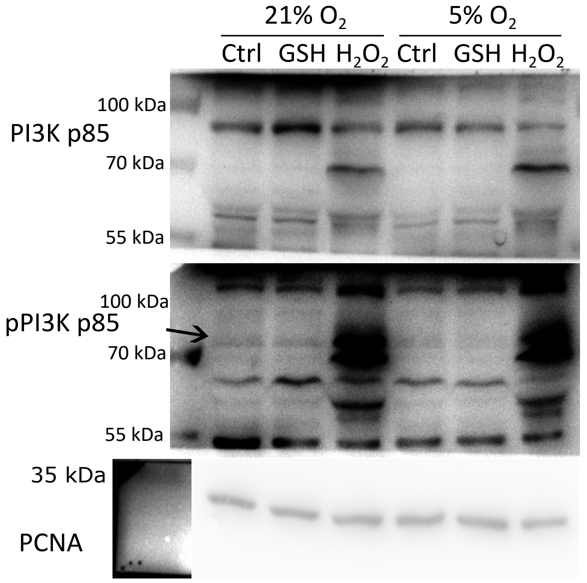

Figure 5A

Membranes were developed using Immobilon Western Chemiluminiscent HRP Substrate and SYNGENE G:Box Chemi

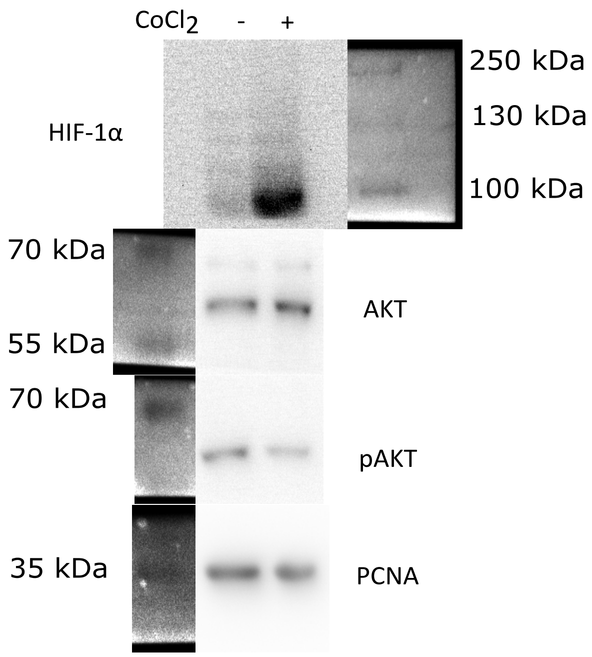

Figure 5B

Membranes were developed using Immobilon Western Chemiluminiscent HRP Substrate and SYNGENE G:Box Chemi

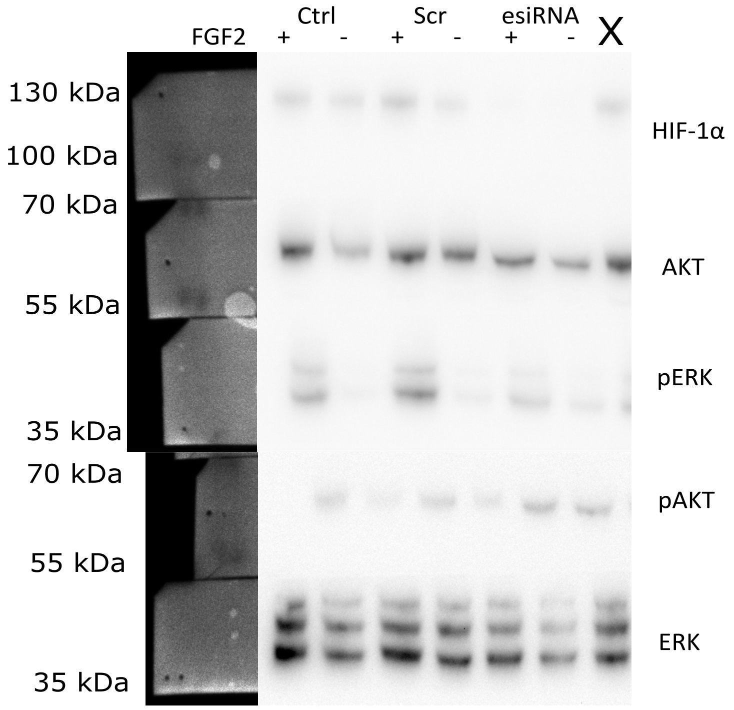

Figure 6A

Membranes were developed using Immobilon Western Chemiluminiscent HRP Substrate and SYNGENE G:Box Chemi

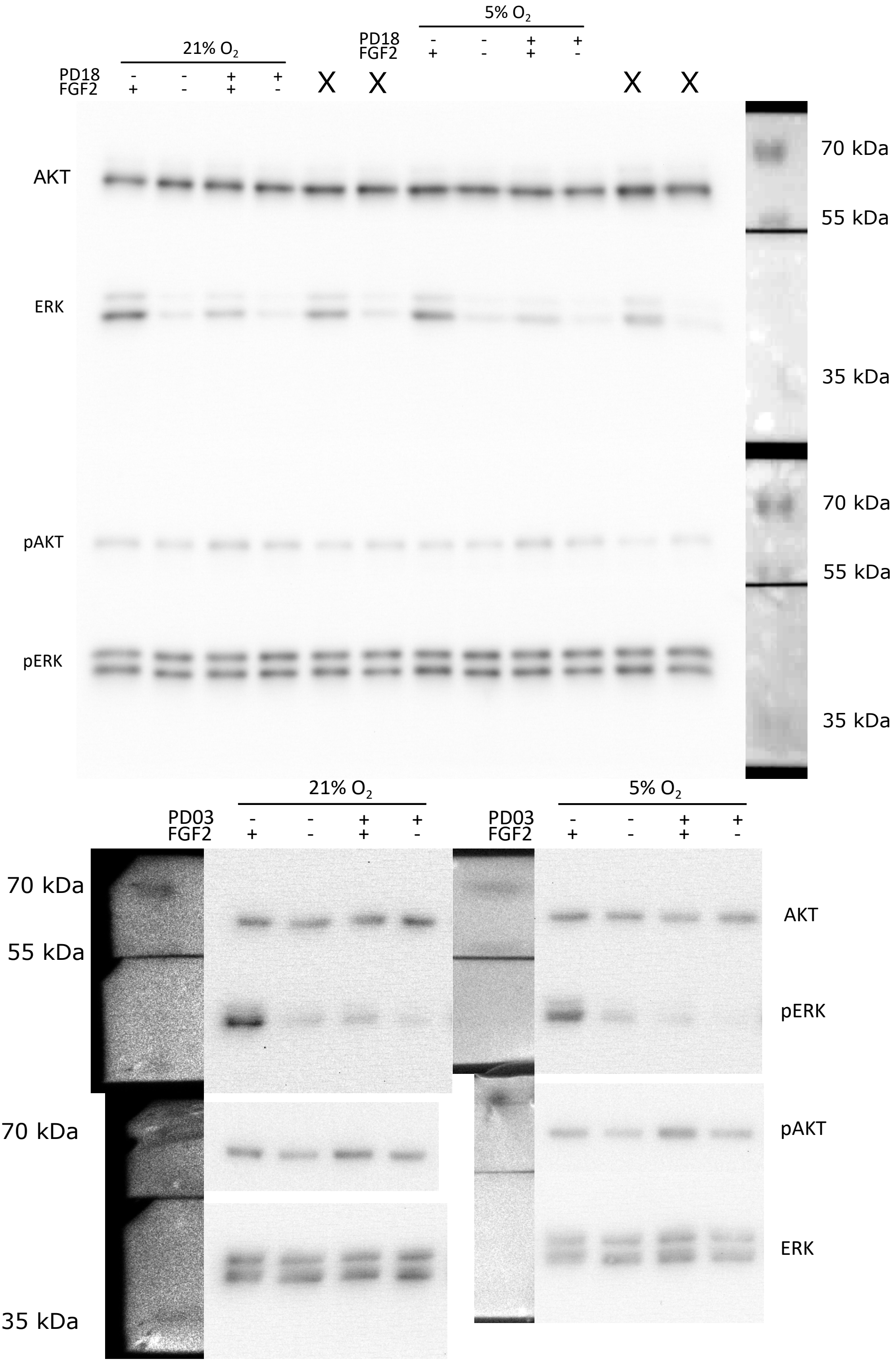

Figure 6C

Membranes were developed using Immobilon Western Chemiluminiscent HRP Substrate and SYNGENE G:Box Chemi

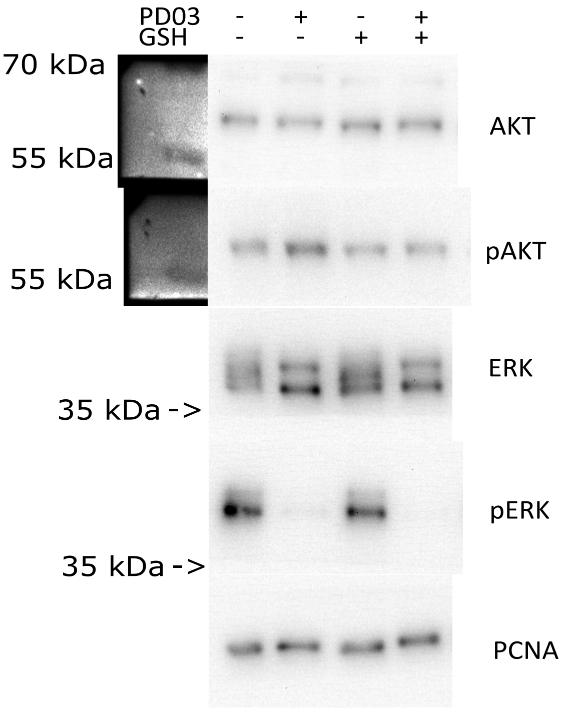

Figure 6D

Membranes were developed using Immobilon Western Chemiluminiscent HRP Substrate and SYNGENE G:Box Chemi

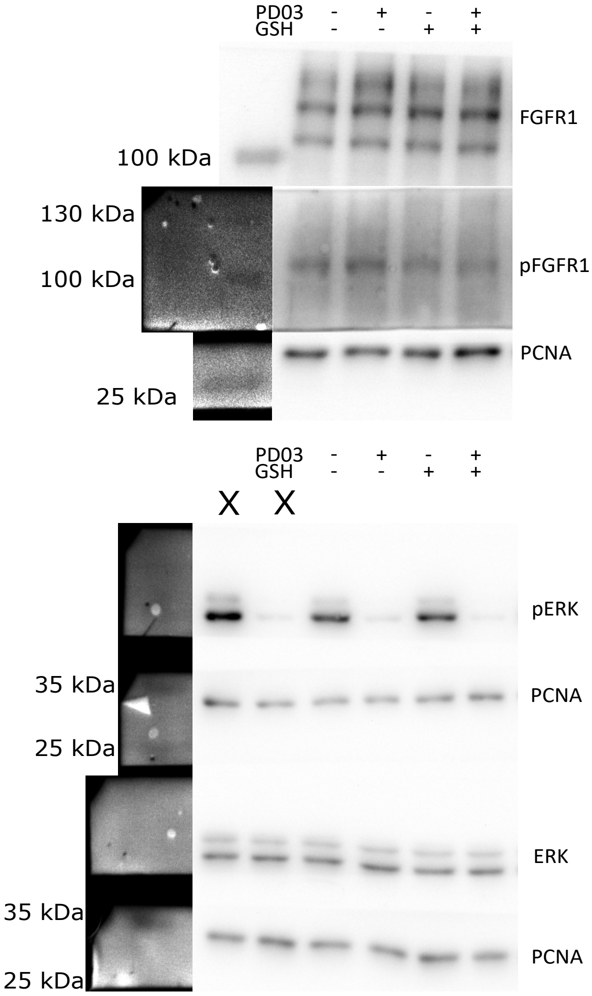

Figure 7A

Membranes were developed using Immobilon Western Chemiluminiscent HRP Substrate and SYNGENE G:Box Chemi

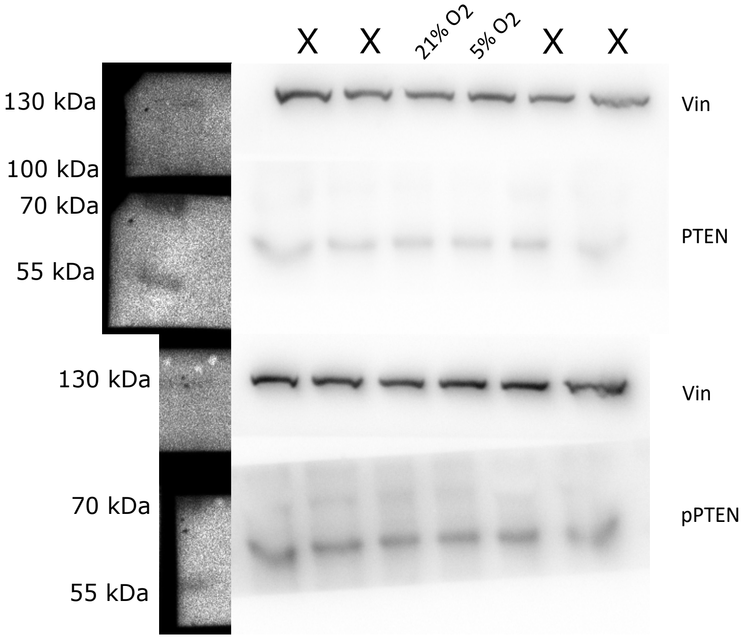

Figure 7B

Membranes were developed using Immobilon Western Chemiluminiscent HRP Substrate and SYNGENE G:Box Chemi

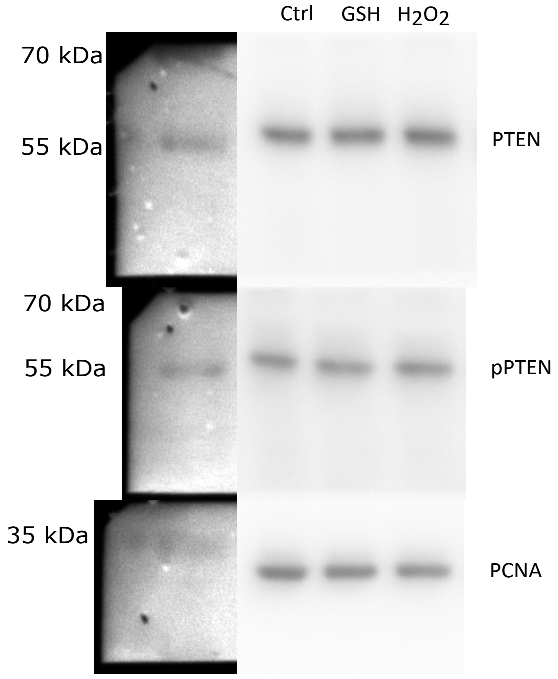

Figure 7C

Membranes were developed using Immobilon Western Chemiluminiscent HRP Substrate and SYNGENE G:Box Chemi

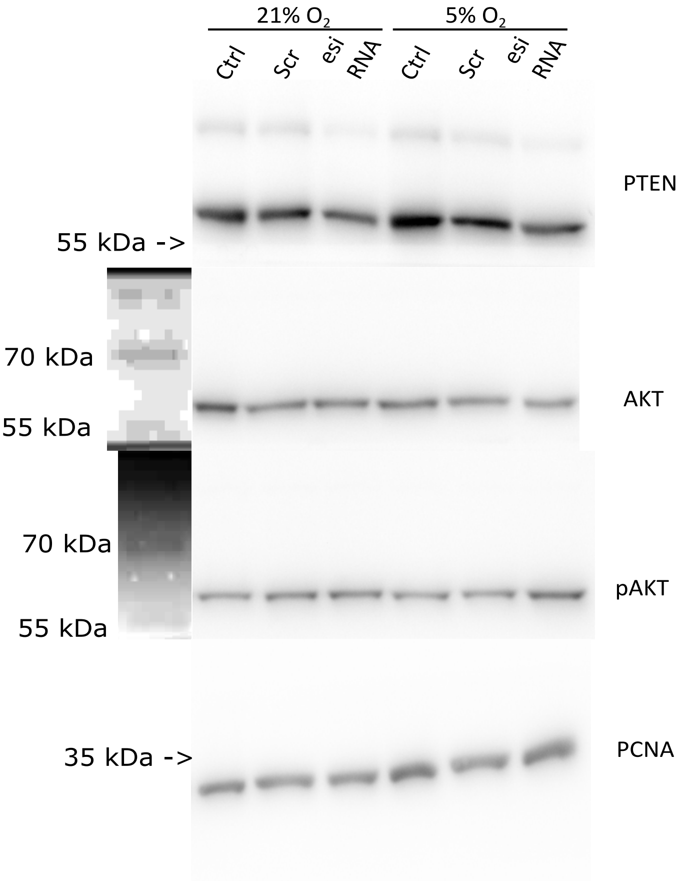

Figure 7E

Membranes were developed using Immobilon Western Chemiluminiscent HRP Substrate and SYNGENE G:Box Chemi

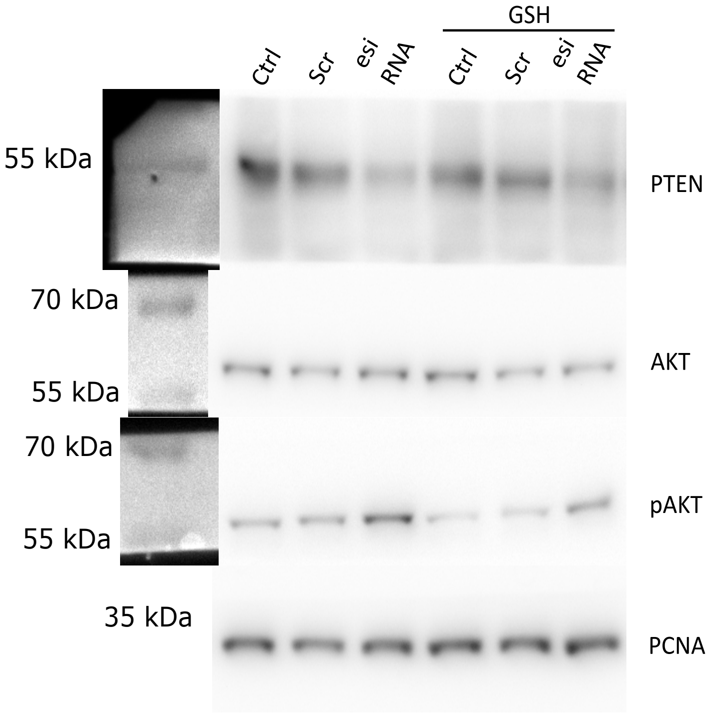

Figure 8A

Membranes were developed using Immobilon Western Chemiluminiscent HRP Substrate and SYNGENE G:Box Chemi

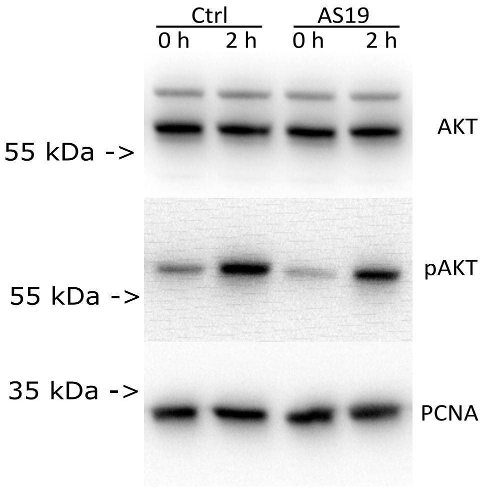

Figure 8B

Membranes were developed using Immobilon Western Chemiluminiscent HRP Substrate and SYNGENE G:Box Chemi

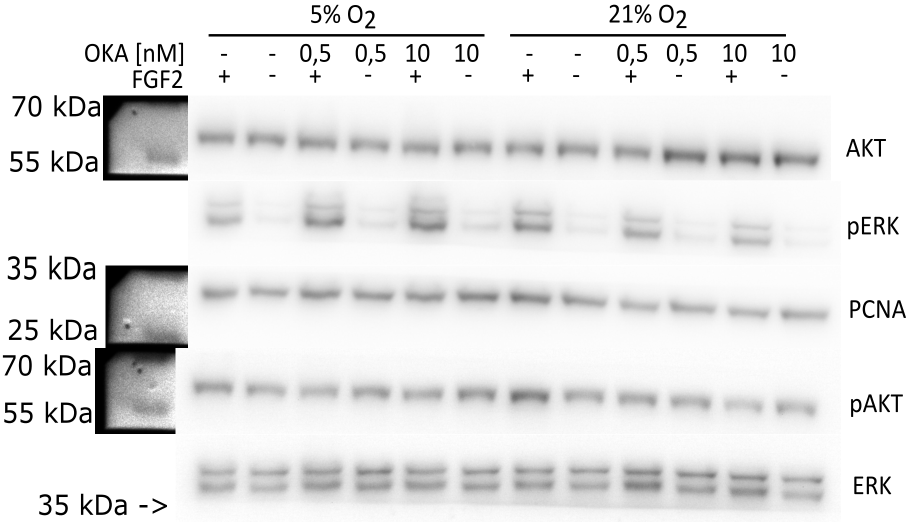

Supplement: Supplementary file 1 [file Presentation_1.PDF]
