# Supplementary figures and images for: Both Hypoxia-Inducible Factor 1 and MAPK Signaling Pathway Attenuate PI3K/AKT via Suppression of Reactive Oxygen Species in Human Pluripotent Stem Cells
Source: Front Cell Dev Biol. 2021 Jan 21;8:607444. doi: 10.3389/fcell.2020.607444 (PMC7859355; doi:10.3389/fcell.2020.607444)

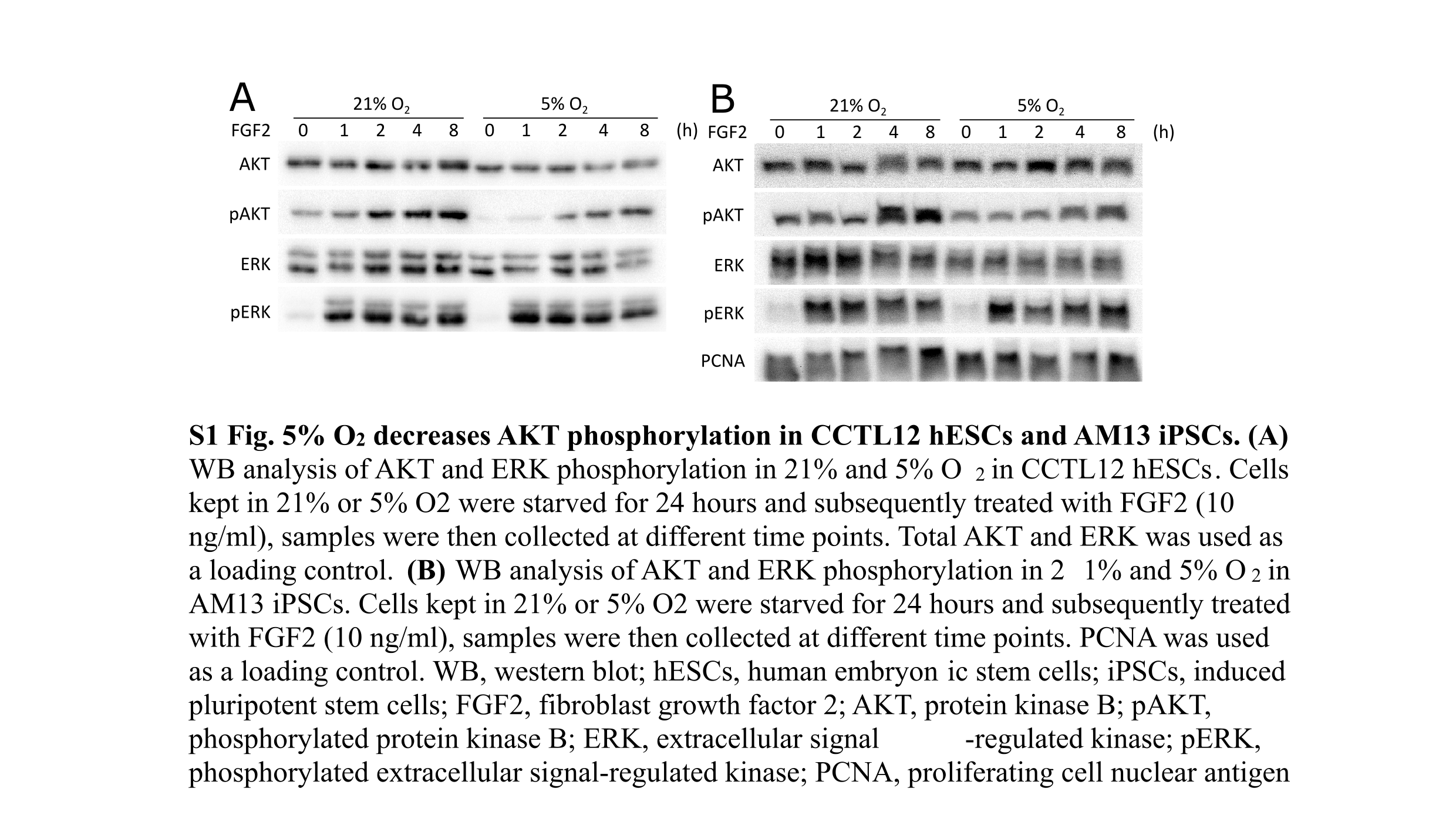

Supplement: Supplementary file 3 [file Image_1.TIF]

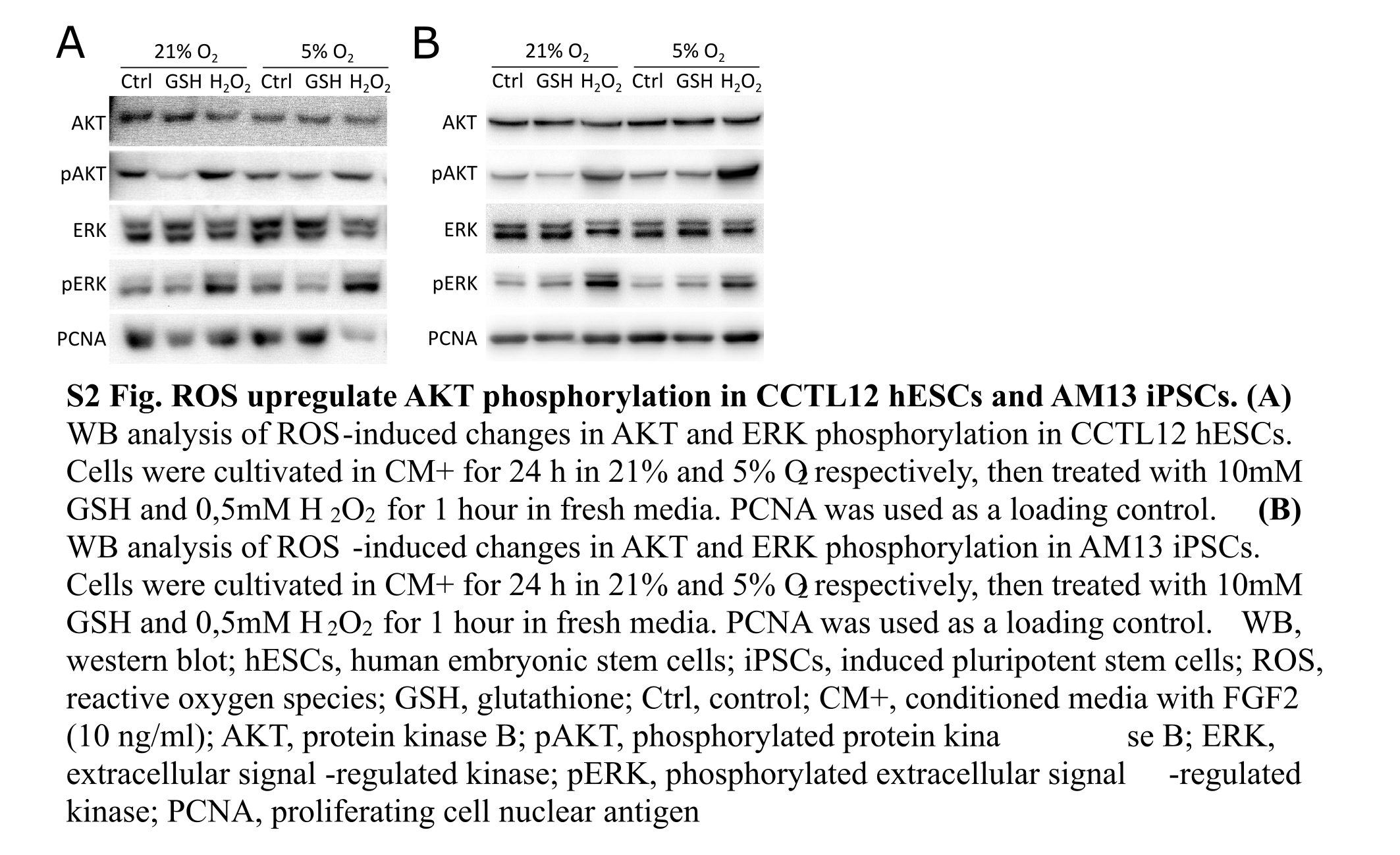

Supplement: Supplementary file 4 [file Image_2.TIF]

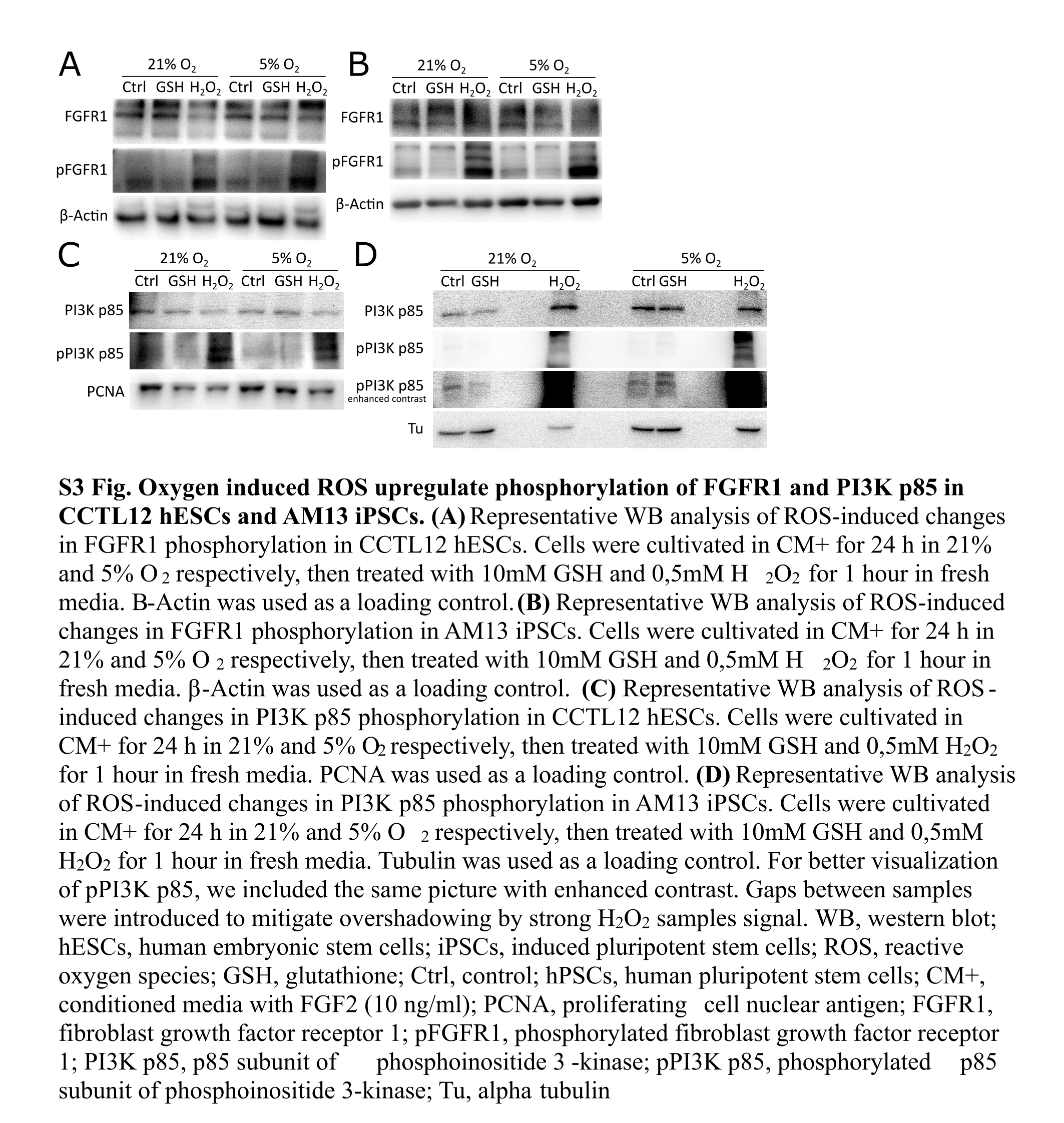

Supplement: Supplementary file 5 [file Image_3.TIF]

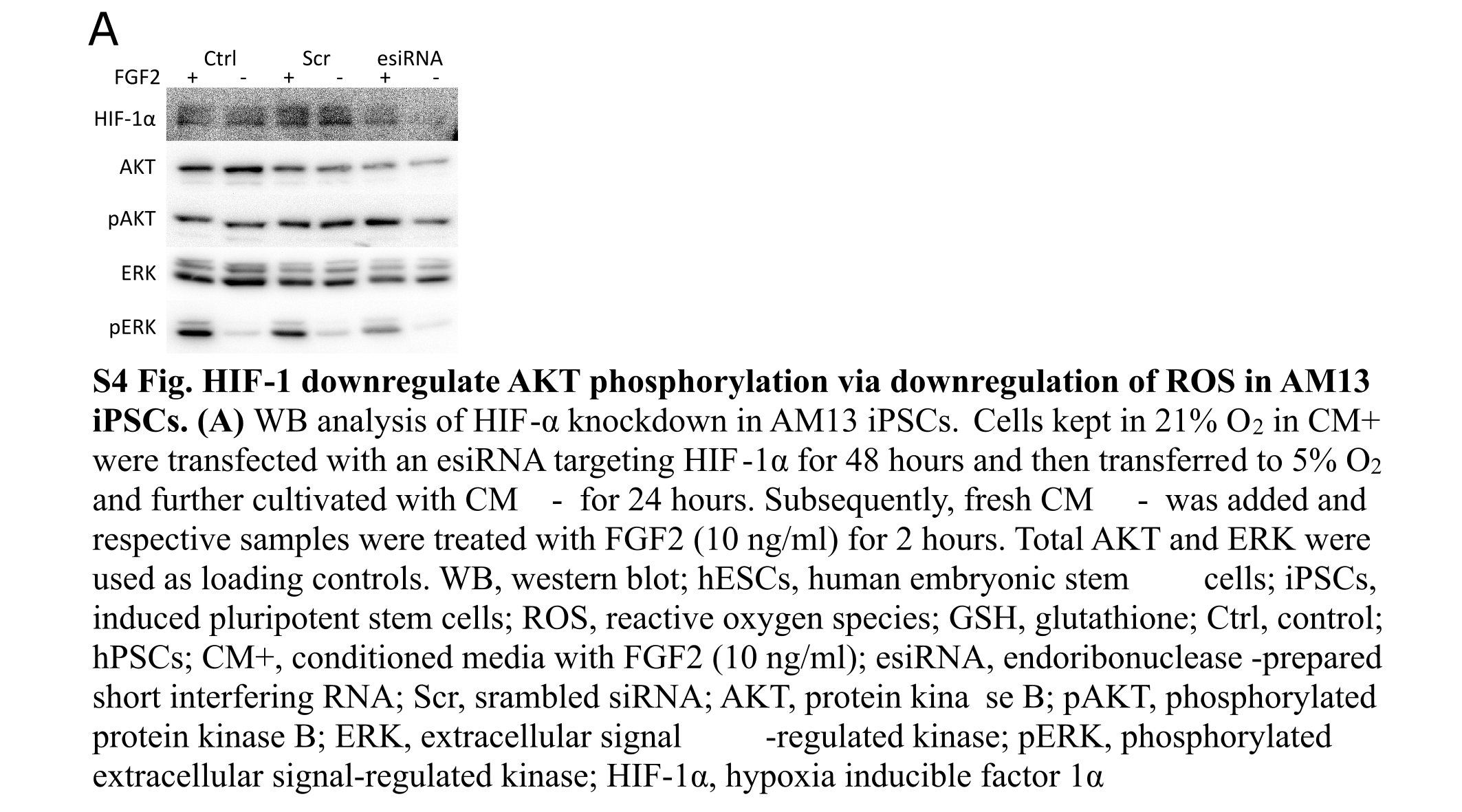

Supplement: Supplementary file 6 [file Image_4.TIF]

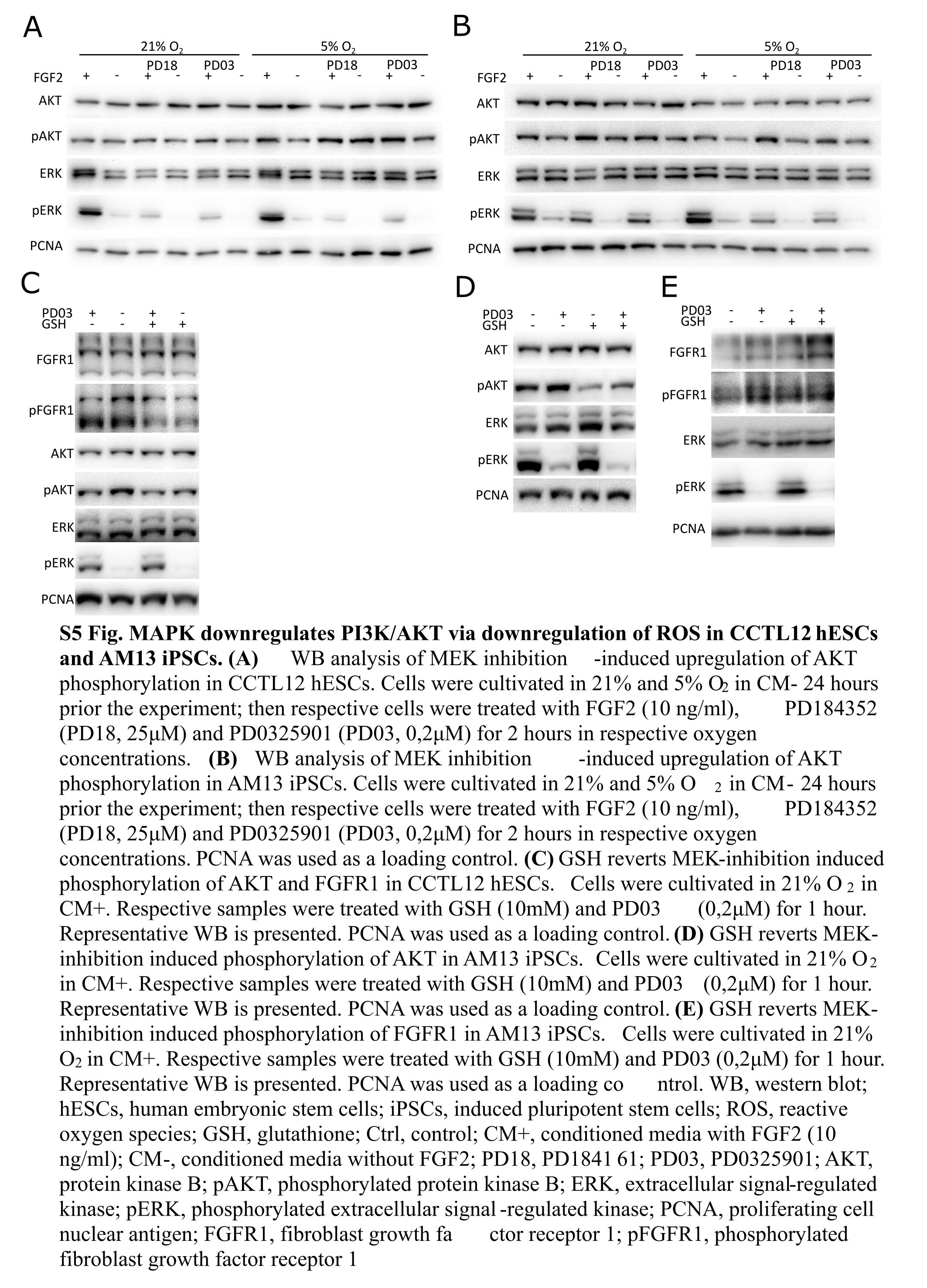

Supplement: Supplementary file 7 [file Image_5.TIF]

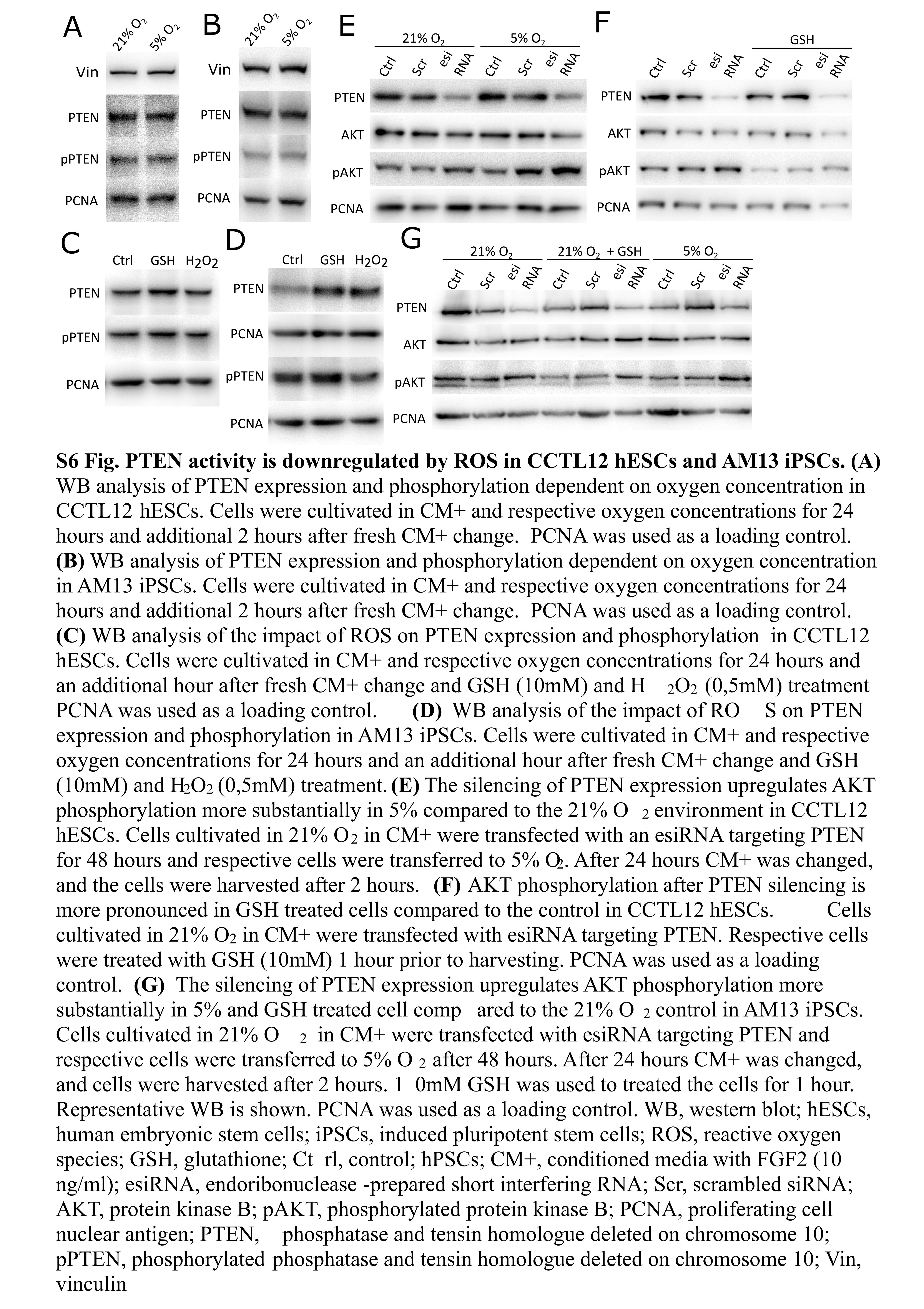

Supplement: Supplementary file 8 [file Image_6.TIF]

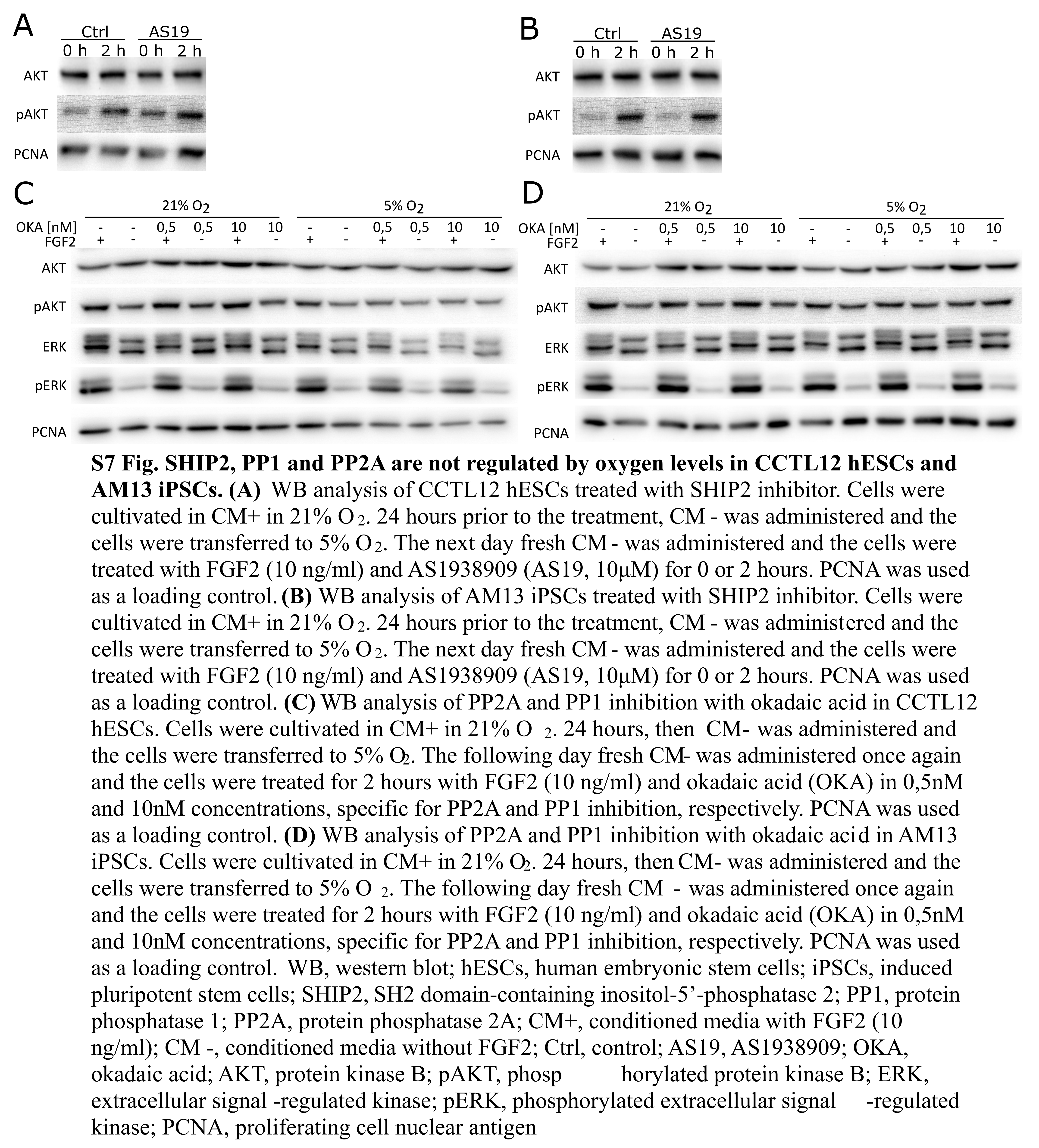

Supplement: Supplementary file 9 [file Image_7.TIF]

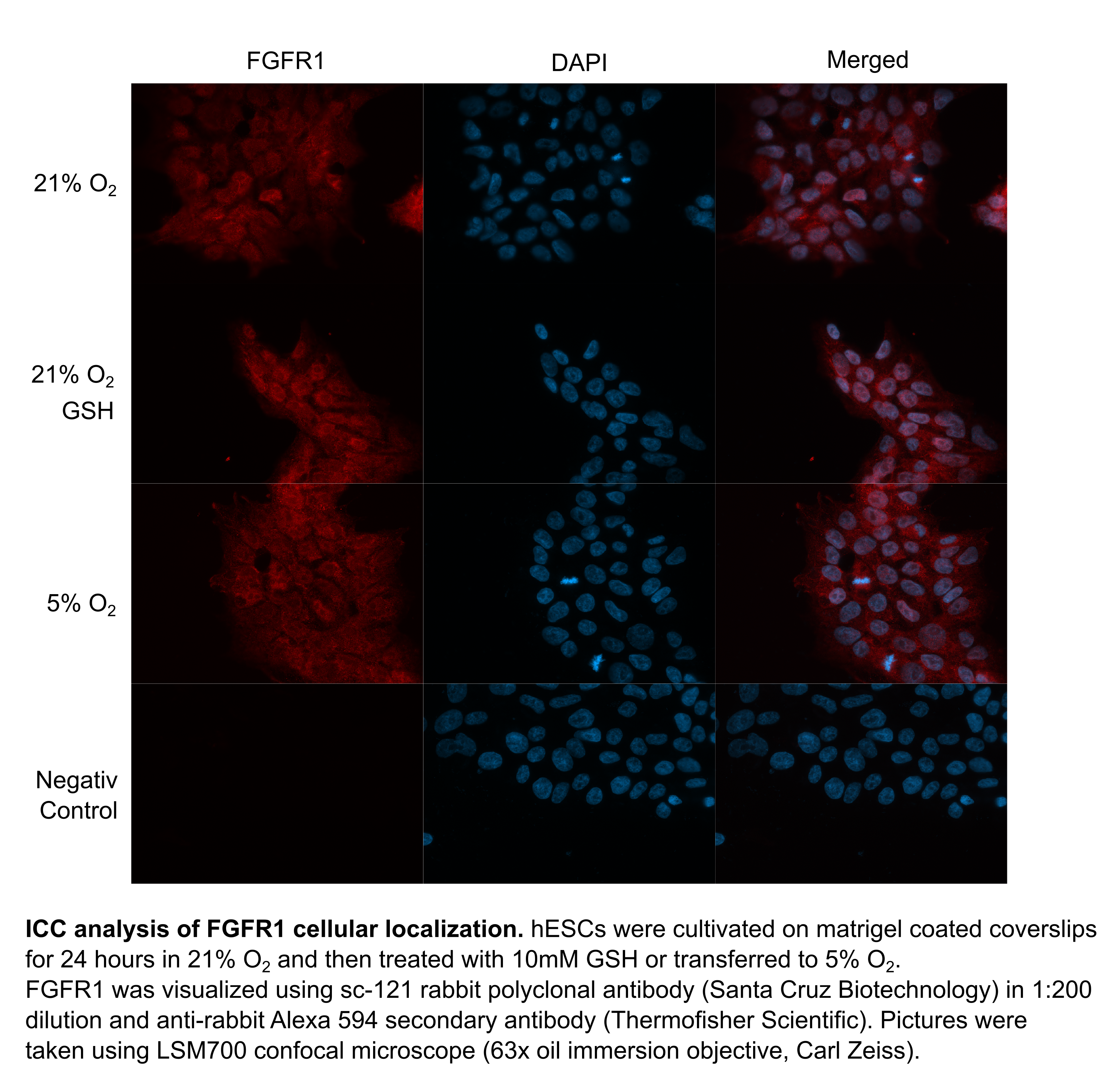

Supplement: Supplementary file 10 [file Image_8.TIF]
